# Unravelling the neuroprotective mechanisms of carotenes in differentiated human neural cells: Biochemical and proteomic approaches

**DOI:** 10.1016/j.fochms.2022.100088

**Published:** 2022-02-14

**Authors:** Kasthuri Bai Magalingam, Sushela Devi Somanath, Nagaraja Haleagrahara, Kanga Rani Selvaduray, Ammu Kutty Radhakrishnan

**Affiliations:** a Jeffrey Cheah School of Medicine and Health Sciences, Monash University Malaysia, Bandar Sunway, Malaysia; b Pathology Division, School of Medicine, International Medical University, Kuala Lumpur, Malaysia; c College of Medicine and Dentistry, James Cook University, Townsville, Queensland, Australia; d Development and Advisory Services, Malaysian Palm Oil Board, Kajang, Malaysia

**Keywords:** 6-OHDA, 6-hydroxydopamine, AD, Alzheimer’s disease, BCM, beta-carotene-15,15′-monooxygenase, CAT, catalase, DRD2, dopamine receptor D2, ER, endoplasmic reticulum, GO, gene ontology, HSP, Heat shock protein, HSPA9, Heat shock protein family A (HSP70) member 9, HSPD1, Heat shock protein family D (HSP60) member 1, KEGG, Kyoto Encyclopedia of Genes and Genomes, LC-MS/MS, liquid chromatography-double mass spectrometry, LDH, lactate dehydrogenase, MCODE, minimal common oncology data elements, MS, mass spectrometry, PD, Parkinson's disease, PDI, protein disulphide isomerases, PHB2, prohibitin 2, PPI, protein–protein interaction, RAN, Ras-related nuclear protein, ROS, reactive oxygen species, RPs, ribosomal proteins, SOD, superoxide dismutase, TH, tyrosine hydroxylase, TMC, total mixed carotene complex, Mixed carotene, 6-hydroxydopamine, SH-SY5Y neuroblastoma cells, Dopamine

## Abstract

Carotenoids, fat-soluble pigments found ubiquitously in plants and fruits, have been reported to exert significant neuroprotective effects against free radicals. However, the neuroprotective effects of total mixed carotenes complex (TMC) derived from virgin crude palm oil have not been studied extensively. Therefore, the present study was designed to establish the neuroprotective role of TMC on differentiated human neural cells against 6-hydroxydopamine (6-OHDA)-induced cytotoxicity. The human neural cells were differentiated using retinoic acid for six days. Then, the differentiated neural cells were pre-treated for 24 hr with TMC before exposure to 6-OHDA. TMC pre-treated neurons showed significant alleviation of 6-OHDA-induced cytotoxicity as evidenced by enhanced activity of the superoxide dismutase (SOD) and catalase (CAT) enzymes. Furthermore, TMC elevated the levels of intra-neuronal dopamine and tyrosine hydroxylase (TH) in differentiated neural cells. The 6-OHDA induced overexpression of α-synuclein was significantly hindered in neural cells pre-treated with TMC. In proteomic analysis, TMC altered the expression of ribosomal proteins, α/β isotypes of tubulins, protein disulphide isomerases (PDI) and heat shock proteins (HSP) in differentiated human neural cells. The natural palm phytonutrient TMC is a potent antioxidant with significant neuroprotective effects against free radical-induced oxidative stress.

## Highlights

• Total mixed carotenes (TMC) protect differentiated human neural cells against 6-hydroxydopamine-induced toxicity.
• TMC elevated the antioxidant enzymes activities and suppressed generation of reactive oxygen species.
• TMC augmented the dopamine and tyrosine hydroxylase levels.
• TMC exerted differential protein expression in human neural cells.

## 1 Introduction

Carotenoids are orange, yellow and red pigments found in plants and microorganisms like algae, fungi and bacteria. These tetraterpenoids, which are C40 hydrocarbons with isoprenoids as building units, can be divided into two groups based on their polarity, i.e. xanthophylls (polar) and carotene (non-polar) (Park, Hayden, Bannerman, Jansen, & Crowe-White, 2020). The polar xanthophylls such as astaxanthin, β-cryptoxanthin, lutein and zeaxanthin are commonly located across lipid bilayer membranes, while the non-polar carotenoids such as α-carotene, β-carotene and lycopene usually reside in the inner part of a cell membrane (Augustynska, 2015) (Widomska, Zareba, & Subczynski, 2016). The daily recommended dietary intake (RDI) of the three primary forms of carotenoids are: α-carotene more than 0.6 mg/day, β-carotene more than 4.0 mg/day and lutein more than 3.3 mg/day (Institute of Medicine (US) Panel on Dietary Antioxidants and Related Compounds, 2000).

The human brain, particularly the frontal cortex, is reported to be rich in sixteen carotenoids such as anhydrolutein, α-carotene, α-cryptoxanthin, β-carotene, β-cryptoxanthin, lycopene, lutein and zeaxanthin; and an age-related decline in total carotenoids was observed in the frontal lobe (Craft, Haitema, Garnett, Fitch, & Dorey, 2004). In addition, low levels of α-carotene, β-carotene and lycopene were detected in the serum of patients with Parkinson’s disease (PD) with advanced stage compared to those with early disease onset (Kim et al., 2017). In this regard, the low level of serum carotenoids in PD patients may be caused by increased uptake of carotenes by brain neuronal cells rather than lacking them in the first place. Therefore, carotenes are potent antioxidants that may contribute to the longevity of neurons in the human brain and carotene deficiency may contribute to the development of neurodegenerative disease. A great deal of attention has been committed to delineating the molecular actions of carotene on neuronal cells, and one long-standing theory is its antioxidant effects against free radicals.

The reactive oxygen species (ROS) scavenging activity of β-carotene has been demonstrated using the highly sensitive and selective chemiluminescence pyrogallol-luminol and luminol-hydrogen peroxide (H_2_O_2_) assays (Chang, Chang, & Lai, 2013). A recent report showed that the administration of β-carotene provided neuroprotective effects in a controlled cortical impact model by suppressing the ROS levels, which reduced brain oedema following a traumatic brain injury (Chen et al., 2019). This study revealed that β-carotene augmented cognitive performance and neural functions by modulating the Nrf2/Keap1-mediated antioxidant pathway.

Lycopene, the non-polar derivative of carotene provides vegetables and fruits’ red-pink colour. Some of the beneficial activities of lycopene include superior bioavailability (Basu & Imrhan, 2006), heat stability during food processing (Colle, Lemmens, Van Buggenhout, Van Loey, & Hendrickx, 2010) and the ability to cross the blood-brain-barrier (Guo et al., 2019). Several neurodegenerative diseases are caused by dysregulation in Ca^2+^ signalling that impairs neurons’ electrical potential and synaptic loss (Pchitskaya, Popugaeva, & Bezprozvanny, 2018). However, a proper dietary intake of lycopene, β-carotene and astaxanthin have been shown to improve Ca^2+^ homeostasis in the brain (Rzajew, Radzik, & Rebas, 2020). Furthermore, Huang et al have demonstrated that lycopene protects neural stem cells by preventing apoptosis by inhibiting expression of Bax/Bcl-2, cytochrome *C* and caspase 3 and reducing intracellular build-up of hydrogen peroxide, oxidative stress and lipid peroxidation. This study also suggested that lycopene may be capable of stimulating nerve growth factor (NGF), brain-derived neurotrophic factor (BDNF) and vascular endothelial growth factor (VEGF) from neural stem cells (Huang et al., 2018).

It is crucial to explore the cellular and molecular changes implicated by carotenes on human neural cells to better undertand the neuroprotective actions of these bioactive compounds. The proteomic approach using label-free mass spectrometry (MS) has enormous potential to provide a robust basis of the molecular mechanism on how natural phytonutrients modulate protein expressions in various cellular processes, including metabolism, respiration, cytoskeletal organisation, protein folding and ribosome biogenesis. The protein network interaction and pathway analysis of the altered biological, molecular and signalling pathways can be acquired through a statistical framework integrated with online bioinformatics tools such as Metascape (Zhou, Zhou, Pache, Chang, Khodabakhshi, Tanaseichuk, Benner, & Chanda, 2019), Kyoto Encyclopedia of Genes and Genomes (KEGG) (Kanehisa, 2019) and DAVID TOOL (Wu, Hasan, & Chen, 2014). In the present study, we investigated the neuroprotective actions of TMC on differentiated SH-SY5Y human neural cells in relation to antioxidant activity, alteration in dopamine biosynthesis, expression of the *dopamine receptor 2 (DRD2)* gene as well as identification of proteins that are differentially expressed in these cells following exposure to TMC.

## 2 Materials and methods

### 2.1 Materials

#### 2.1.1 Total mixed carotene extract

The palm fruit-derived total mixed carotene (TMC) extract used in this study contained α-, β-, γ-carotenes and lycopene. It was a kind gift from ExcelVite Sdn. Bhd (Chemor, Malaysia).

#### 2.1.2 Reagents and instrument

Dulbecco’s Modified Eagle Media (DMEM) was purchased from Corning Incorporated (NY, USA), fetal bovine serum (FBS) was obtained from Biosera (Nuaille, France), non-essential amino acid (NEAA) and penicillin-streptomycin (P/S) were purchased from Thermo Fisher Scientific Inc. (San Jose, USA) and 6-OHDA, levodopa, retinoic acid and WST cell proliferation (WST-1 reagent Cat. No. 11644807001) were purchased from MilliporeSigma (Burlington, USA). The LDH assay kit (Cat. No. 601170), ROS Detection Cell-Based Assay Kit (Cat. No. 601520), SOD assay kit (Cat. No. 706002) and Catalase Assay kit (Cat. No. 707002) were procured from Cayman chemical (Ann Arbor, USA). The Dopamine ELISA Kit assay kit (Cat. No. KA3838) was obtained from Abnova (Taipei city, Taiwan), ELISA Kit for Tyrosine Hydroxylase (Cat. No. SEB438Hu) was acquired from Cloud-Clone Corp (Katy, USA) and α-Synuclein (SNCA) (Human) assay kit (Cat. No. K4261) was bought from BioVision Incorporated (Milpitas, USA). The RNeasy® Plus Mini Kit (Cat. No. 74134) was obtained from Qiagen (Hilden, Germany), 2x qPCRBIO SyGreen 1-Step Mix and 20x RTase with RNase inhibitor (Cat. No. PB25.11-01) was procured from PCR Biosystems (London, UK) and human primers: All-in-One™ qPCR Primer for Human DRD2 [NM_016574.2] and All-in-One™ qPCR Primer for Human GAPDH [NM_002046.6] were acquired from Genecopoeia Inc (Rockville, USA). The EasyPrep Mini MS Sample Prep kit (Cat. No. A40006) was purchased from Thermo Fisher Scientific Inc. (San Jose, USA). The absorbance was taken using the SpectramaxM microplate reader that was obtained from Molecular Devices (San Jose, USA), The CT values were generated from the iQ5 Optical Module PCR Detection System provided by Bio-Rad (Hercules, USA) and the proteomic data were collected from the Agilent 1200 HPLC-Chip/MS Interface connected with Agilent 6550 iFunnel Q-TOF LC/MS provided by Agilent (Santa Clara, USA).

### 2.2 Methods

#### 2.2.1 Differentiation and culture of the SH-SY5Y human neuroblastoma cells

The SH-SY5Y human neuroblastoma cells (CRL-2266, ATCC, VA, USA) were cultured in DMEM supplemented with 10% FBS, 1% NEAA and 1% P/S at 37 °C in a humidified atmosphere of 5% carbon dioxide. The SH-SY5Y cells were differentiated to matured homogenous dopaminergic neurons as previously described (Magalingam, Radhakrishnan, Somanath, Md, & Haleagrahara, 2020). Briefly, the SH-SY5Y neuroblastoma cells were adjusted to 1x10^5^ cells/mL in complete medium and seeded in various culture flasks at 37 °C in a humidified 5% CO_2_ incubator for 24 hr. The cell density was varied based on the culture vessels used for the different assays. For instance, for 96-well plates, 100 μL/well of the 1x10^5^ cells/mL cell suspension was used, making it 1x10^4^ cells/well; for 6-well plates, 4 mL of cell suspension was used (4x10^5^ cells/well); for T75 flask, 10 mL of cell suspension was used (1x10^6^ cells/flask). After 24 hr, the culture medium was replaced with a differentiating medium, which was similar to the culture medium except that it contained low serum (3% FBS) and 10 μM retinoic acid. The SH-SY5Y cells were maintained in the differentiating medium for three (3) days. The spent medium was replaced on the fourth day, and the plates/culture vessels were returned to the incubator. The cells were allowed to differentiate for 6 days. On day 7, prominent changes on the SH-SY5Y neuroblastoma cells that resemble human dopaminergic neural cells were observed. These cells were used to investigate the neuroprotective effect of TMC against 6-OHDA -induced toxicity. For cell viability and lactate dehydrogenase (LDH) assays, 96-well plates were used and for the antioxidant and dopamine biosynthesis assays, 6-well plates were used. Finally, the cells were cultured in T75 flasks for gene expression and proteomic studies.

#### 2.2.2 Experimental protocol

The differentiated SH-SY5Y neural cells were pre-treated with TMC (0.1 μg/mL) for 24 h at 37 °C in a humidified 5% CO_2_ incubator. The TMC concentration of 0.1 μg/mL was selected based on the preliminary cytotoxicity assessment, which used a wider range of TMC concentrations (0–20 μg/mL) (data not shown). After 24 h of exposure to TMC, the neurotoxin 6-OHDA was added to the differentiated neural cells. This chemical induces selective cytotoxic damage on human neural cells. The neuroprotective potential of the TMC on the differentiated SH-SY5Y neural cells was evaluated using various biochemical assays. Levodopa (0.1 μg/mL), a standard drug used in the treatment of PD, was included in this study as a drug control.

In proteomics assay, the differentiated SH-SY5Y neural cells were treated with TMC or 6-OHDA for 24 h, and total protein was extracted from the cell lysate and digestion prior to analysis using the liquid chromatography with double mass spectrophotometry (LC-MS/MS). The alteration in the protein expression following TMC or 6-OHDA treatment on the differentiated SH-SY5Y neural cells was compared with the protein expression in untreated control cells.

#### 2.2.3 Biochemical study

##### 2.2.3.1 Cell viability assay

The differentiated SH-SY5Y human neural cell were pre-treated with TMC at 37 °C for 24 hr in a humidified 5% CO_2_ incubator before they were exposed to 6-OHDA. To perform the cell viability assay, 10 μL of the WST-1 cell proliferation reagent was added to each well. The culture plate was mixed thoroughly for 1 min using an orbital shaker and returned to the incubator for 2 hr. The developed formazan dye was quantified by measuring the absorbance at the wavelength of 450 nm (reference wavelength of 630 nm) using a spectrophotometer. The percentage (%) of viable cells was calculated using the formula shown below:

**Table:** 

Absorbance of test – background*	X	100%
Absorbance of negative control – background*		
*Background: blank i.e. well with no cells		

##### 2.2.3.2 Lactate dehydrogenase assay

The differentiated SH-SY5Y cells were pre-treated with TMC for 24 hr and subsequently exposed to 6-OHDA for another day. For this experiment, wells with 20 μL of Triton X-100 (provided with the kit) and 20 μL of assay buffer (provided with the kit) were included as controls. At the end of the treatment period, the culture supernatant was harvested by centrifugation (1500 g for 10 min at 4 °C) and stored at −80 °C prior to analysis. To perform the LDH assay, 100 μL of the sample was placed in each 96-well plate with 100 μL of LDH Reaction Solution (provided with the kit). Then, the culture plate was incubated for 30 min on an orbital shaker. The absorbance was read at 490 nm using the microplate reader. The Triton X-100 treated wells were regarded as “maximum release” and other test samples as “spontaneous release samples”. The background reading was subtracted from all wells and the % cytotoxicity was calculated as follows:

% Cytotoxicity of test sample =

**Table:** 

(Experimental Value A490) – (Spontaneous release)	X	100%
(Maximum Release A490) – (Spontaneous release)		
* Background was subtracted from all wells		

##### 2.2.3.3 Reactive oxygen species assay

The SH-SY5Y neuroblastoma cells were seeded on a black tissue culture-treated 96-well plate, differentiated and treated according to the experimental set-up. The ROS assay was performed at the end of the treatment procedure using the ROS Detection Cell-Based Assay Kit. Briefly, the culture medium was aspirated from the 96-well culture plate, and ROS staining buffer (provided with the kit) was added to the wells. Then, the plate was incubated for 30 min at 37 °C. Following the incubation, the ROS staining buffer was aspirated, and cell-based assay buffer (provided with the kit) was added to the wells. The intensity of the fluorescence dye was measured using 490 nm excitation and 525 nm emission wavelengths using the microplate reader.

##### 2.2.3.4 Superoxide dismutase assay

The TMC treated neural cells were homogenized, and cell lysates were prepared. As recommended in the SOD assay kit, the cell lysates were mixed with the diluted radical detector (provided with the kit). The reaction was triggered by adding the diluted xanthine oxidase (provided with the kit) with the precise time taken. The plate was incubated on a shaker for 30 min at room temperature, and the absorbance was recorded at 450 nm using the microplate reader.

##### 2.2.3.5 Catalase assay

Cell lysates were prepared for all treated samples as recommended in the Catalase Assay kit. The experiment was performed by mixing the cell lysates with diluted Assay buffer and methanol *(provided with the kit)*. The peroxidatic reaction was triggered by adding the hydrogen peroxide *(provided with the kit)* and subsequently incubating the plate for 20 min on a shaker. Then, the reaction was ceased by adding the potassium hydroxide followed by the addition of the catalase purpald *(provided with the kit)* and the plate was incubated for 10 min. Finally, catalase potassium periodate *(provided with the kit)* was added and the plate was incubated for 5 min. The colorimetric changes were quantitated at 540 nm wavelength using the plate reader.

##### 2.2.3.6 Dopamine assay

The dopamine level in homogenized neuronal cell lysates was estimated using the Dopamine ELISA Kit assay kit in three key steps – extraction, acylation and enzymatic assay. In the extraction step, cell lysates were placed into the respective wells of the extraction plate (provided with the kit). Ultrapure water and TE buffer (provided with the kit) were added to the respective wells, and the plate was incubated for 60 min at room temperature (RT) on a shaker (approx. 600 rpm). Following the incubation, the plate was thoroughly rinsed using wash buffer (provided with the kit) and blotted to remove the remnants of the buffer. In the acylation step, Acylation buffer (provided with the kit) and Acylation reagent (provided with the kit) were added to the extraction plate and incubated for 15 min. Then, the plate was vigorously washed with wash buffer (provided with the kit) and blotted before adding hydrochloric acid into the wells and incubated for 10 min on a shaker. The acylated samples (from the extraction plate) were transferred into the 96-well microtiter plate, followed by adding enzyme solution. Next, the microtiter plate was incubated for 2 h at 37 °C on a shaker. Then, the supernatant was transferred into the pre-coated dopamine microtiter strips (provided with the kit). The dopamine antiserum (provided with the kit) was added into the wells before incubating for 15–20 h at 2–8 °C. Following the incubation step, the plate was thoroughly rinsed with wash buffer (provided with the kit). After the washing step, enzyme conjugate (provided with the kit) was pipetted into the wells and incubated for 30 min on a shaker. The plate was washed and blotted before adding substrate (provided with the kit) and further incubated for 25 min on a shaker. Finally, the stop solution (provided with the kit) was added, and absorbance of dye development was measured using a microplate reader at 450 nm with reference wavelength at 630 nm.

##### 2.2.3.7 Tyrosine hydroxylase assay

The tyrosine hydroxylase (TH) assay was performed as instructed in the ELISA Kit for Tyrosine Hydroxylase. Briefly, the homogenized cell lysates were added into the pre-coated strip plate accordingly and incubated for 1 h at 37 °C. Subsequently, the plate was emptied by blotting the plate before adding the detection reagent A (provided with the kit) and incubated for 1 h at 37 °C. Then, the strip plate was thoroughly rinsed before adding the reagent B (provided with the kit), and the plate was incubated for 30 min at 37 °C. Following this, a substrate solution (provided with the kit) was added and further incubated for 20 min at 37 °C. Finally, the stop solution (provided with the kit) was added, and the development of the dye was estimated at 450 nm using the microplate reader.

##### 2.2.3.8 Alpha synuclein assay

The level of alpha-synuclein in treated differentiated SH-SY5Y neural cells was estimated using the α-Synuclein (SNCA) (Human) assay kit. Firstly, culture supernatants were added into the ELISA plate (provided with the kit) and incubated for 1.5 h at 37 °C. Following the incubation, the plate was rinsed with 1X wash solution (provided with the kit), added with biotin-detection antibody (provided with the kit) and incubated at 37 °C for 60 min. After the incubation, the plate was washed thoroughly with 1X wash solution (provided with the kit). The horseradish peroxidase-streptavidin conjugate (SABC) (provided with the kit) was added into the wells and the plate was incubated for 30 min at 37 °C. Subsequently, the plate was vigorously washed with 1X wash solution (provided with the kit) before adding the 3,3′,5,5′-Tetramethylbenzidine or TMB substrate (provided with the kit). Next, the plate was further incubated for 30 min. Finally, the stop solution (provided with the kit) was added, and the colour development was quantitated at 450 nm using a microplate reader.

##### 2.2.3.9 DRD2 gene expression

The treated differentiated SH-SY5Y neural cells were isolated and RNA extraction was performed according to the manufacturer’s instruction in the RNeasy® Plus Mini Kit. The quality and concentration of the extracted RNA were estimated via the NanoQuantTM technique using the spectrophotometer. The RNA samples were stored at −80 °C prior to real-time (RT)-PCR analysis. The RT-PCR analysis was performed on 20 μL of reagent volume consisting of 10 pg of RNA template prepared in PCR grade distilled water, 2 ng/μL All-in-One™ qPCR Primer for Human DRD2, 2x qPCRBIO SyGreen 1-Step Mix and 20x RTase with RNase inhibitor. For normalization of RT-PCR data, All-in-One™ qPCR Primer for Human GAPDH gene was included as the housekeeping gene. The instrument condition was set at 40 cycles of denaturation at 95 °C for 5 s, followed by annealing and extension steps, each at 60 °C for 30 s. All experiments were performed in triplicates. A dissociation curve analysis was performed at the end of the 40 cycles to ascertain the amplification specificity using the iQ5 Optical Module PCR Detection System. The difference in gene expression of the DRD2 (target gene [TG]) and GAPDH (reference gene [RG]) was compared between TMC + 6-OHDA (target sample [TS]) and 6-OHDA alone (reference sample [RS]) using the formulae: 2^^(−ΔΔCt)^ {ΔCt = Ct[TG] – Ct[RG]; ΔΔCt = ΔCt[TS] – ΔCt[RS]} (Rao, Huang, Zhou, & Lin, 2013)

#### 2.2.4 Proteomic study

##### 2.2.4.1 Protein extraction, reduction and alkylation

After the treatment protocol, cells were harvested and recovered by centrifugation (1000 g for 10 min at 4 °C). The pellet was collected, and the total protein extraction was performed using the EasyPrep Mini MS Sample Prep kit. Briefly, lysis buffer (provided with the kit) and universal nuclease (provided with the kit) were added to the cell pellets and mixed until the sample's viscosity was lessened. The extracted protein samples were stored at − 80 °C until further use. In the protein reduction step, the protein samples were thawed and 100 µg of protein was transferred into microtubes. A final volume for each sample was adjusted to 100 µL with lysis solution (provided with the kit). Then, a reduction solution (provided with the kit) was added and mixed gently. Following this, alkylation solution (provided with the kit) was added to the tubes. These tubes were incubated at 95 °C using a heat block for 10 min to allow the reduction and alkylation reactions to occur. Then, samples were cooled and subjected to Trypsin/Lys-C protein digestion procedure.

##### 2.2.4.2 Protein digestion and clean-up

The enzyme for the digestion step was prepared by mixing the enzyme reconstitution solution (provided with the kit) with Trypsin/Lys-C-Protease mix (provided with the kit). Subsequently, the reconstituted Trypsin/Lys-C-Protease mix was added to each tube containing the samples. The tubes were incubated at 37 °C with shaking for 3 h to allow protein digestion to occur. Following the 3 h, the digestion stop solution (provided with the kit) was added to each tube to terminate the digestion process. After the peptide digestion, peptide cleaning was carried out using the peptide clean-up column (provided with the kit) to eliminate any contaminants present in the samples. The digested peptides were transferred into the peptide clean-up column and centrifuged. The flow-through from the column was discarded. Next, wash solution A (provided with the kit) was added into the column and centrifuged with flow-through discarded after the spin. This step was repeated using wash solution B (provided with the kit). Finally, the column was placed on sterile collection tubes, and elution solution (provided with the kit) was added to each column. The eluted peptide samples were collected by centrifugation, dried using a vacuum centrifuge and stored at −80 °C before LC-MS/MS analysis.

##### 2.2.4.3 Liquid chromatography and mass spectrometry analysis

The Trypsin/Lys-C-Protease digested peptides were loaded into the Agilent 1200 HPLC-Chip/MS Interface connected with Agilent 6550 iFunnel Q-TOF LC/MS. The column was equilibrated with 0.1% formic acid in water (solution A). The peptides were eluted from the column with 90% acetonitrile in 0.1% formic acid in water (solution B). Quadrupole-time of flight (Q-TOF) polarity was set at positive with capillary and fragmenter voltage at 1900 V and 360 V, respectively, and 5 L/min of gas flow with a temperature of 325 °C. The collision energy was determined at 3.7 V (100 Da), and reference masses with positive polarity was set at 299.294457 and 1221.990637. The peptide spectrum was analysed in auto MS mode ranging from 110 to 3000 *m*/*z* for MS scan and 50–3000 *m*/*z* for MS/MS scan.

##### 2.2.4.4 Data calculation and bioinformatic analysis

The raw data were obtained and processed using PEAKS X software (Bioinformatics Solutions Inc., Waterloo, ON, Canada) using Uniprot, Swissprot and TrEMBL databases. The PEAKS X software allows for the determination of the protein abundance using the following search parameters: retention time lower bound: ≥ 0, retention time upper bound: ≤ 55, average area: ≥ 0, charge lower bound: ≥ 1, confident number samples per group: ≥ 1, peptide identification count: ≥ 1, protein significance: ≥ 20, used peptides: ≥ 1, fixed modification: Carbamidomethylation of cysteine residues and false discovery rate (FDR): 1% in three biological replicate injections. The obtained peptide/protein list was exported to Microsoft Excel for further downstream computation. Proteins expressed in at least 2 biological samples were filtered out, while proteins that only existed in one biological replicate were eliminated from the data set. The TMC or 6-OHDA implicated proteins were compared independently with proteins regulated in untreated SH-SY5Y neural cells. Proteins expressing statistically significant (p<0.05) differences were identified and further scrutinized for function-relevant gene annotations and protein-protein interaction networks using the Metascape tool (https://metascape.org/gp/index.html). The TMC and 6-OHDA implicated significant protein sets were imputed into the Metascape interface with the default parameter set. The process and pathway enrichment analysis for each protein set were carried out using the gene ontology sources, including GO Biological Processes, KEGG pathway, Reactome Gene Sets, Canonical Pathways, PANTHER Pathway CORUM and WikiPathways. According to Metascape analysis, all genes in the genome have been used as the enrichment background. Terms with a p < 0.01, a minimum count of 3, and an enrichment factor >1.5 (the enrichment factor is the ratio between the observed counts and the counts expected by chance) are collected and grouped into clusters based on their membership similarities. More specifically, p-values are calculated based on the accumulative hypergeometric distribution, and q-values are calculated using the Benjamini-Hochberg procedure to account for multiple tests. Kappa scores are used as the similarity metric when performing hierarchical clustering on the enriched terms, and sub-trees with a similarity of >0.3 are considered a cluster. The most statistically significant term within a cluster is chosen to represent the cluster. In order to capture the relationships between the terms, a subset of enriched terms have been selected and rendered as a network plot, where terms with a similarity >0.3 are connected by edges. We selected the terms with the best p-values from each of the 20 clusters, with the constraint that there are no more than 15 terms per cluster and no more than 250 terms in total. Subsequently, the protein-protein interaction enrichment analysis for each protein set (6-OHDA or TMC) was executed using STRING, BioGrid, OmniPath, InWeb_IM databases. The generated PPI network contains the subset of proteins that form physical interactions with at least one other member in the list. If the network contains between 3 and 500 proteins, the Molecular Complex Detection (MCODE) algorithm has been applied to identify densely connected network components. The independent MCODE component analysis was performed, and the three best-scoring terms by p-values have been identified as the functional description of the corresponding component. The Metascape analysis enabled the identification of significantly overexpressed functional protein clusters, PPI network and PPI MCODE components (unit) associated with PD.

##### 2.2.4.5 Venn diagram analysis

We cross-checked with the gene sets present in PD databases to identify the important TMC modulated PD associated genes in the set of 87 differentially regulated genes. For this objective, we queried three PD-specific databases, namely: Beegle, a disease-gene discovery database that narrows down the high-probability of disease-causing genes obtained from gene sequencing, linkage analysis and association studies (http://beegle.esat.kuleuven.be/) (ElShal et al., 2016); DisGeNet, a discovery platform that contains collections of data from expert-curated repositories, GWAS catalogues, animal model and scientific literature (https://www.disgenet.org/) (Piñero et al., 2017); Gene4PD, an integrative genomic database and analytic platform for PD (http://genemed.tech/gene4pd/home). Using the keyword “(Parkinson’s disease)”, a total of 194 PD-associated genes were retrieved from Beegle, 1515 genes from DisGeNet and 3222 genes from Gene4PD databases. Then, we matched the differentially regulated genes by TMC on neural cells to the PD associated genes in these three databases. The TMC implicated genes that matched the PD-causing genes in these databases were identified, and a Venn diagram was constructed to illustrate the overlapping of these genes. A histogram was also constructed to visualize some of the genes/proteins that exhibited a significant (p < 0.05) difference in expression between 6-OHDA and TMC treatments.

#### 2.2.5 Statistical analysis

For the biochemical study, the experiments were repeated three times in triplicates and data from one experiment was shown as mean ± SEM. The differences between the treatment groups were determined using one-way ANOVA, followed by Bonferroni post-hoc test. In proteomic data analysis, differentially expressed proteins implicated by TMC or 6-OHDA were compared with untreated neural cells using a two-tailed Student's t-test. The analysis for biochemical and proteomic studies was conducted using SPSS Ins. Software (SPSS Statistics, V22.0.0). A value of p < 0.05 was considered statistically significant.

## 3 Results

### 3.1 TMC enhanced differentiated human neural cells viability in 6-OHDA induced toxicity

**Fig. 1:**
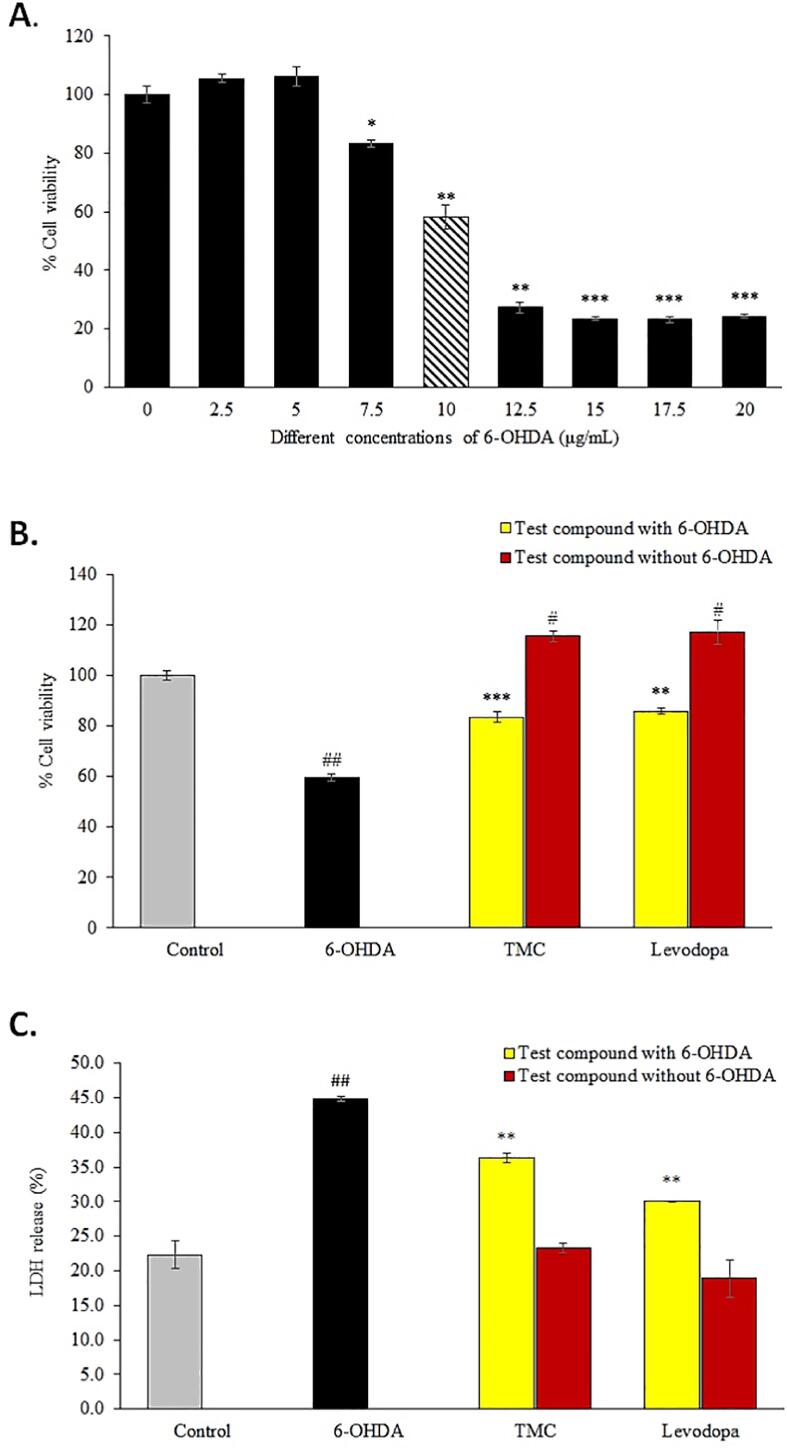
**(A)** The effects of exposing the differentiated SH-SY5Y neural cells to different concentrations of 6-OHDA (0–20 μg/mL) for 24 hr to identify the IC50 concentration of 6-OHDA. Cell viability was determined using WST-1 cell proliferation assay and is expressed as mean ± SEM (n = 3). *p < 0.05, **p < 0.01, ***p < 0.001 (6-OHDA treatment (2.5–20 μg/mL) vs untreated control. The effects of pre-treating the differentiated SH-SY5Y neural cells to TMC (0.1 μg/mL) for 24 hr prior exposure to IC50 concentration of 6-OHDA (10 μg/mL) for a further 24 hr **(B)** cell viability analysis and **(C)** LDH release. In both **(B)** and **(C)**, pre-treatment with levodopa (0.1 μg/mL) was included as positive control; while cells pre-treated with just culture medium served as negative control. Data are expressed as mean ± SEM (n = 3). ^#^p < 0.05, ^##^p < 0.01 (6-OHDA vs control & compound alone vs control); **p < 0.01, ***p < 0.001 (TMC + 6-OHDA vs 6-OHDA & levodopa + 6-OHDA vs 6-OHDA).

Before investigating the neuroprotective effect of TMC, we evaluated the dose-response of 6-OHDA on differentiated SH-SY5Y neural cells to identify the concentration of 6-OHDA that induces approximately 50% cell death. After 24 h exposure with 6-OHDA, there was a marked reduction in cell viability compared to untreated control. The decline in cell viability, which started at 7.5 μg/mL 6-OHDA, peaked around 15.0 μg/mL 6-OHDA (Fig. 1A). The concentration of 6-OHDA that caused 50% cell death or IC50 (58.23 ± 4.0; p < 0.01) was determined to be 10 μg/mL (Fig. 1A). Hence, this concentration of 6-OHDA was used in the subsequent assays. We also assessed the effects of treating the differentiated SH-SY5Y cells with different concentrations (0.01 μg/mL to 20 μg/mL) of TMC and levodopa (data not shown) and from these preliminary studies, we identified that a low concentration (0.1 μg/mL) of TMC or levodopa provided neuroprotective effects within 24 h of the pre-treatment. There was a marked reduction in the viability of the differentiated SH-SY5Y neural cells when exposed to 6-OHDA [59.8% ± 1.4, (p < 0.05)] compared to untreated cells (Fig. 1B). However, pre-treatment with TMC prior to exposure to 6-OHDA, caused a significant increase in cell viability [83.52% ± 1.1 (p < 0.001)] (Fig. 1B). A similar increase in cell viability was observed when the cells were pre-treated with levodopa (85.8% ± 1.1) prior to exposure to 6-OHDA (Fig. 1B). In addition, pre-treatment with TMC or levodopa prior to exposure to 6-OHDA, cause a marked decrease (p < 0.01) in the levels of LDH produced (Fig. 1C).

### 3.2 TMC suppressed ROS generation and augmented antioxidant enzymes

**Fig. 2:**
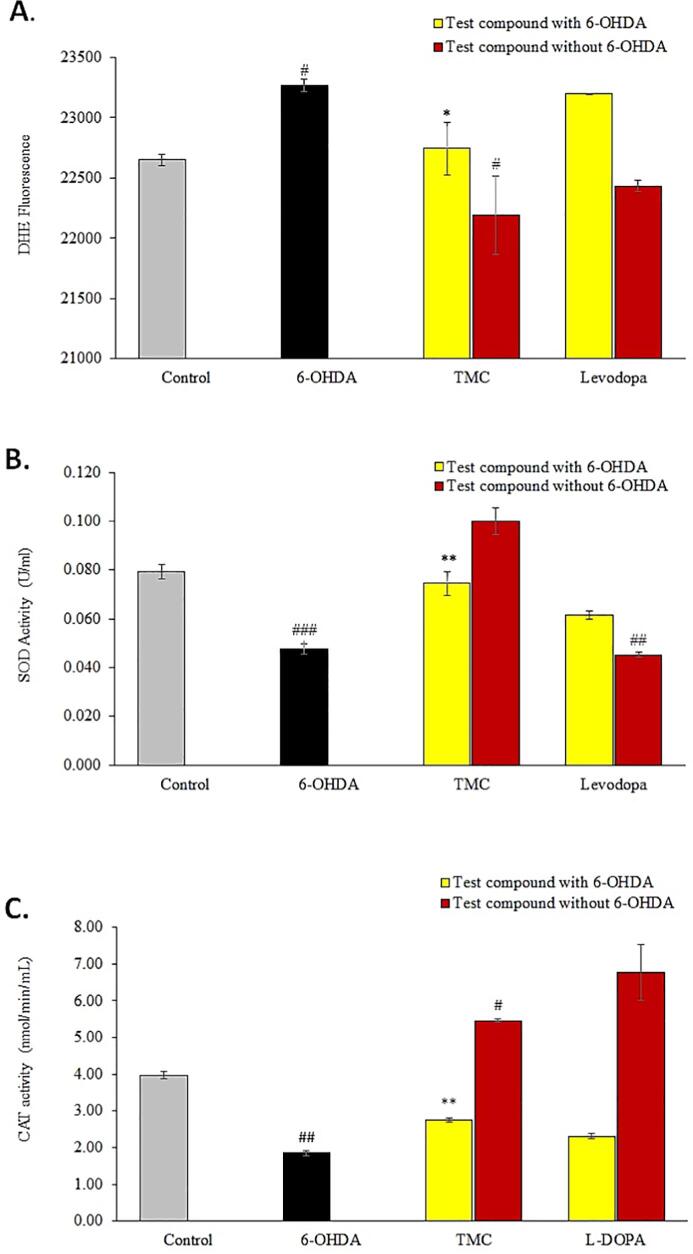
Alterations in **(A)** ROS generation, **(B)** SOD levels and **(C)** CAT levels in the differentiated SH-SY5Y neural cells following pre-treatment with TMC (0.1 μg/mL) or levodopa (0.1 μg/mL) prior to exposure to 6-OHDA (10 μg/mL). The effect of treating the differentiated SH-SY5Y cells with TMC (0.1 μg/mL) or levodopa (0.1 μg/mL) in the absence of 6-OHDA are included for comparison. Pre-treatment with levodopa was included as positive control; while cells pre-treated with just culture medium served as negative control. Results are shown as mean ± SEM (n = 3). ^#^p < 0.05, ^##^p < 0.01, ^###^p < 0.001 (6-OHDA vs control & test compound alone vs control]; *p < 0.05, **p < 0.01 (TMC + 6-OHDA vs 6-OHDA).

To study the antioxidant effects of TMC, we examined the ROS generation (Fig. 2A) and activities of two endogenous antioxidant enzymes, namely SOD (Fig. 2B) and CAT (Fig. 2C). Our study showed a marked upsurge in ROS generation by the differentiated SH-SY5Y neural cells exposed to 6-OHDA for 24 hr compared to untreated cells. However, pre-treating neuronal cells with TMC for 24 hr prior to exposure to 6-OHDA caused a significant reduction in ROS generation with *p < 0.05*. The treatment of differentiated SH-SY5Y neural cells with 6-OHDA for 24 hr resulted in a marked decline in SOD and CAT enzyme activities compared to the untreated control group. However, the SOD and CAT enzyme activities were significantly increased in differentiated SH-SY5Y pre-treated with TMC *(p < 0.01)* prior to treatment with 6-OHDA compared to 6-OHDA alone group.

### 3.3 TMC altered dopamine, TH, α-synuclein levels and DRD2 mRNA fold change in differentiated human neural cells

**Fig. 3:**
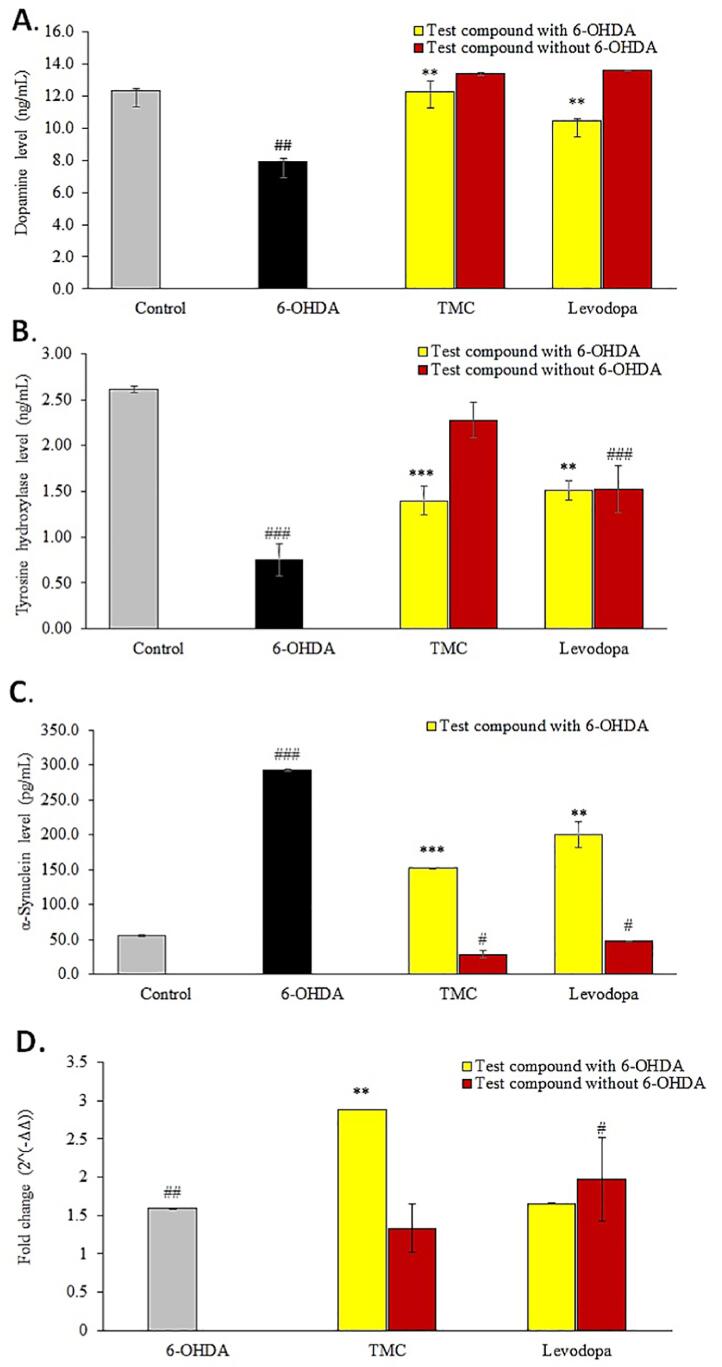
The effects of treating the differentiated SH-SY5Y neural cells to TMC (0.1 μg/mL) in the absence and presence of exposure to 6-OHDA (10 μg/mL) on **(A)** dopamine levels; **(B)** TH levels; **(C)** α-synuclein levels and **(D)** expression of the *DRD2* mRNA fold change. The levels of dopamine, TH and α-synuclein were quantified using commercial kits using the respective manufacturer recommended protocol while the expression of the DRD2 gene was determine using quantitative polymerase chain reaction (qPCR). Pre-treatment with Levodopa was included as positive control; while cells pre-treated with just culture medium served as negative control. Data are expressed as mean ± SEM (n = 3). ^#^p < 0.05, ^##^p < 0.01, ^###^p < 0.001 (6-OHDA vs control & treatment compound vs control); **p < 0.01, ***p < 0.001 (TMC + 6-OHDA vs 6-OHDA).

To evaluate the influence of TMC on the dopamine biosynthesis pathway, we quantitated the levels of dopamine (Fig. 3A), TH (Fig. 3B) and α-synuclein (Fig. 3C) produced by the differentiated SH-SY5Y cells treated 6-OHDA in the absence or presence of TMC treatment. Our study revealed that exposure of human neural cells to 6-OHDA significantly attenuated the intraneuronal dopamine and TH levels but overexpressed the α-synuclein level. However, pre-treating the differentiated human neural cells with TMC for 24 hr prior to 6-OHDA exposure, caused a significant elevation in dopamine (p < 0.01) and TH *(p < 0.001)* levels when compared to cells treated with 6-OHDA alone. Moreover, TMC also blocked the 6-OHDA induced overexpression of α-synuclein (p < 0.001) from these cells. Similar neuroprotective effects were also observed with levodopa pre-treatment and subsequent 6-OHDA exposure on dopamine, TH and α-synuclein levels. The expression of the DRD2 mRNA fold change in the differentiated SH-SY5Y cells treated with 6-OHDA in the absence or presence of TMC or levodopa was analysed using quantitative real time PCR assay. The gene expression results were normalized with the Ct values of the *GAPDH* housekeeping gene (Fig. 3D). Pre-treatment with TMC increased the *DRD2* mRNA fold change [2.88 ± 0.0 (p < 0.01)] in differentiated SH-SY5Y neural cells compared to cells treated with 6-OHDA (1.60 ± 0.0). In contrast, no significant change in DRD2 fold change was noted in differentiated neural cells pre-treated with levodopa.

### 3.4 Label-free mass spectrophotometry quantification of differentially expressed protein

The alteration in protein expression following 24 hr exposure of differentiated neural cells to 6-OHDA (10 μg/mL) or TMC (0.1 μg/mL) was performed using label-free tandem liquid mass spectrometry (LC-MS/MS). These proteins were compared against untreated differentiated SH-SY5Y neural cells. The PEAKS X + software was used to filter the data according to the set criteria. Only proteins with at least one confidence peptide with false discovery rate (FDR) less than or equal to 0.01 (FDR ≤ 0.01) in all three biological replicates per treatment condition were included in analysis.

A total of 3636 proteins were identified in the triplicates of differentiated SH-SY5Y neural cells treated with 6-OHDA. Out of these proteins, 687 proteins were common in the three biological replicates of the 6-OHDA treated cells. When compared with the untreated control, there was a total of 188 proteins that were common between 6-OHDA treated and untreated SH-SY5Y neural cells. Among these 188 proteins, 100 proteins displayed a significant difference with p < 0.05 between 6-OHDA treatment and untreated controls: with 61 upregulated and 39 downregulated proteins with p < 0.05. The top three proteins that were markedly upregulated in the differentiated SH-SY5Y neural cells treated with 6-OHDA were histone H2B type 2-F (HIST2H2BF), Macrophage migration inhibitory factor (MIF) and histone H2B type 1-H (HIST1H2BH) while the top three proteins that were down-regulated were histone H2B type 1-N (HIST1H2BN), heat shock protein beta-1 (HSPB1) and Histone H2B type 1-D (HIST1H2BD) (Table 1).

**Table 1:** Proteins that were (A) up or (B) down-regulated proteins in differentiated SH-SY5Y neural cells following exposure to 6-OHDA.

(A) Upregulated proteins					
Accession	Protein description	Gene symbol	Average mass	p value	**Fold change
Q5QNW6	Histone H2B type 2-F	HIST2H2BF	27,840	2.00E-02	2.062
P14174	Macrophage migration inhibitory factor	MIF	24,952	1.33E-02	2.062
Q93079	Histone H2B type 1-H	HIST1H2BH	27,784	1.80E-02	1.925
P12277	Creatine kinase B-type	CKB	85,288	4.29E-02	1.880
P11021	Endoplasmic reticulum chaperone BiP	HSPA5	144,666	3.32E-02	1.696
Q14103	Heterogeneous nuclear ribonucleoprotein D0	HNRNPD	76,868	1.03E-02	1.695
P63104	14-3-3 protein zeta/delta	YWHAZ	55,490	2.31E-03	1.638
P68431	Histone H3.1	HIST1H3A	30,808	3.61E-02	1.539
Q13509	Tubulin beta-3 chain	TUBB3	100,866	3.10E-03	1.479
O15240	Neurosecretory protein VGF	VGF	134,516	5.50E-07	1.425
Q9P0M6	Core histone macro-H2A.2	H2AFY2	80,116	3.38E-03	1.377
P60709	Actin cytoplasmic 1	ACTB	83,474	4.43E-04	1.356
P52565	Rho GDP-dissociation inhibitor 1	ARHGDIA	46,414	2.74E-04	1.343
P24752	Acetyl-CoA acetyltransferase mitochondrial	ACAT1	90,400	9.43E-03	1.268
P08758	Annexin A5	ANXA5	71,874	6.85E-03	1.252
P25705	ATP synthase subunit alpha mitochondrial	ATP5F1A	119,502	5.29E-03	1.240
P57053	Histone H2B type F-S	H2BFS	27,888	7.78E-03	1.233
P14625	Endoplasmin	HSP90B1	184,938	4.45E-02	1.228
P0DP25	Calmodulin-3	CALM3	33,676	5.93E-03	1.208
Q16555	Dihydropyrimidinase-related protein 2	DPYSL2	124,588	2.02E-02	1.198
P06899	Histone H2B type 1-J	HIST1H2BJ	27,808	1.13E-04	1.186
P61978	Heterogeneous nuclear ribonucleoprotein K	HNRNPK	101,952	3.08E-02	1.175
P05141	ADP/ATP translocase 2	SLC25A5	65,704	4.21E-02	1.173
P33778	Histone H2B type 1-B	HIST1H2BB	27,900	2.00E-02	1.156
P45880	Voltage-dependent anion-selective channel protein 2	VDAC2	63,134	3.02E-05	1.133
P60174	Triosephosphate isomerase	TPI1	61,582	3.45E-02	1.126
P49327	Fatty acid synthase	FASN	546,848	2.43E-04	1.126
P06733	Alpha-enolase	ENO1	94,338	1.53E-04	1.122
Q9UJZ1	Stomatin-like protein 2 mitochondrial	STOML2	77,068	3.50E-02	1.116
P06744	Glucose-6-phosphate isomerase	GPI	126,294	2.12E-02	1.116
P30153	Serine/threonine-protein phosphatase 2A 65 kDa regulatory subunit A alpha isoform	PPP2R1A	130,618	4.75E-02	1.108
Q9Y277	Voltage-dependent anion-selective channel protein 3	VDAC3	61,318	7.24E-03	1.104
P21796	Voltage-dependent anion-selective channel protein 1	VDAC1	61,546	1.45E-02	1.103
P27824	Calnexin	CANX	135,136	6.53E-04	1.100
Q9BUF5	Tubulin beta-6 chain	TUBB6	99,714	1.81E-02	1.093
P16104	Histone H2AX	H2AFX	30,290	4.24E-02	1.093
P63261	Actin cytoplasmic 2	ACTG1	83,586	2.54E-02	1.092
P62249	40S ribosomal protein S16	RPS16	32,890	4.78E-02	1.080
P0DP23	Calmodulin-1 OS = Homo sapiens	CALM1	33,676	1.13E-02	1.078
O75367	Core histone macro-H2A.1	H2AFY	79,234	1.13E-02	1.078
P23527	Histone H2B type 1-O	HIST1H2BO	27,812	1.13E-02	1.078
Q71U36	Tubulin alpha-1A chain	TUBA1A	100,272	1.13E-02	1.078
P30041	Peroxiredoxin-6	PRDX6	50,070	1.98E-02	1.076
P40939	Trifunctional enzyme subunit alpha mitochondrial	HADHA	166,000	4.01E-03	1.071
P68032	Actin alpha cardiac muscle 1	ACTC1	84,038	1.19E-02	1.067
P10809	60 kDa heat shock protein mitochondrial	HSPD1	122,110	1.19E-02	1.067
P61604	10 kDa heat shock protein mitochondrial	HSPE1	21,864	1.19E-02	1.067
Q06830	Peroxiredoxin-1	PRDX1	44,220	1.19E-02	1.067
P68363	Tubulin alpha-1B chain	TUBA1B	100,304	1.19E-02	1.067
Q9BVA1	Tubulin beta-2B chain	TUBB2B	99,906	1.19E-02	1.067
P49411	Elongation factor Tu mitochondrial	TUFM	99,084	1.19E-02	1.067
P55072	Transitional endoplasmic reticulum ATPase	VCP	178,644	1.19E-02	1.067
P07195	L-lactate dehydrogenase B chain	LDHB	73,278	4.58E-04	1.066
P36578	60S ribosomal protein L4	RPL4	95,394	1.59E-03	1.066
P07437	Tubulin beta chain	TUBB	99,342	6.86E-03	1.065
P62841	40S ribosomal protein S15	RPS15	34,080	2.39E-03	1.062
P62851	40S ribosomal protein S25	RPS25	27,484	2.94E-04	1.061
P27797	Calreticulin	CALR	96,284	2.66E-02	1.058
P62081	40S ribosomal protein S7	RPS7	44,254	2.25E-03	1.049
P62753	40S ribosomal protein S6	RPS6	57,362	2.15E-03	1.037
Q15084	Protein disulfide-isomerase A6	PDIA6	96,242	5.39E-03	1.009

(B) Down-regulated proteins					
Accession	Protein description	Gene symbol	Average mass	p value	**Fold change
Q99877	Histone H2B type 1-N	HIST1H2BN	27,844	2.19E-02	0.523
P04792	Heat shock protein beta-1	HSPB1	45,566	1.29E-02	0.573
P58876	Histone H2B type 1-D	HIST1H2BD	27,872	3.09E-02	0.623
P31150	Rab GDP dissociation inhibitor alpha	GDI1	101,166	1.56E-02	0.638
P62805	Histone H4	HIST1H4A	22,734	3.50E-03	0.643
Q99879	Histone H2B type 1-M	HIST1H2BM	27,978	1.97E-02	0.694
Q12905	Interleukin enhancer-binding factor 2	ILF2	86,124	1.97E-02	0.694
P23284	Peptidyl-prolyl cis–trans isomerase B	PPIB	47,486	2.45E-02	0.712
P13639	Elongation factor 2	EEF2	190,676	3.02E-02	0.716
P32119	Peroxiredoxin-2	PRDX2	43,784	1.48E-02	0.730
P00338	L-lactate dehydrogenase A chain	LDHA	73,378	2.52E-02	0.732
P07355	Annexin A2	ANXA2	77,208	1.66E-02	0.734
Q00610	Clathrin heavy chain 1	CLTC	383,226	9.96E-08	0.736
P29373	Cellular retinoic acid-binding protein 2	CRABP2	31,386	9.96E-08	0.736
Q08211	ATP-dependent RNA helicase A	DHX9	281,916	9.96E-08	0.736
Q13263	Transcription intermediary factor 1-beta	TRIM28	177,100	2.66E-02	0.744
P07602	Prosaposin	PSAP	116,226	2.58E-03	0.751
P23246	Splicing factor proline- and glutamine-rich	SFPQ	152,300	3.01E-04	0.752
P0DP24	Calmodulin-2	CALM2	33,676	2.98E-02	0.767
P35232	Prohibitin	PHB	59,608	2.24E-02	0.767
P09936	Ubiquitin carboxyl-terminal hydrolase isozyme L1	UCHL1	49,648	1.03E-03	0.783
P62304	Small nuclear ribonucleoprotein E	SNRPE	21,608	1.22E-03	0.784
P62318	Small nuclear ribonucleoprotein Sm D3	SNRPD3	27,832	8.24E-03	0.789
P06576	ATP synthase subunit beta mitochondrial	ATP5F1B	113,120	7.24E-04	0.792
Q99714	3-hydroxyacyl-CoA dehydrogenase type-2	HSD17B10	53,846	4.07E-03	0.795
P62807	Histone H2B type 1-C/E/F/G/I	HIST1H2BC	27,812	3.38E-02	0.797
P68104	Elongation factor 1-alpha 1	EEF1A1	100,282	1.49E-02	0.809
O60814	Histone H2B type 1-K	HIST1H2BK	27,780	3.98E-02	0.815
P38646	Stress-70 protein mitochondrial	HSPA9	147,362	4.18E-03	0.816
P30049	ATP synthase subunit delta mitochondrial	ATP5F1D	34,980	2.18E-02	0.818
P62829	60S ribosomal protein L23	RPL23	29,730	4.51E-02	0.877
P37802	Transgelin-2	TAGLN2	44,782	6.85E-03	0.878
P62826	GTP-binding nuclear protein Ran	RAN	48,846	6.19E-04	0.883
P46782	40S ribosomal protein S5	RPS5	45,752	2.69E-02	0.883
Q16778	Histone H2B type 2-E	HIST2H2BE	27,840	2.79E-03	0.889
P30101	Protein disulfide-isomerase A3	PDIA3	113,564	4.50E-02	0.912
P19338	Nucleolin	NCL	153,230	4.36E-02	0.926
Q99623	Prohibitin-2	PHB2	66,592	2.65E-03	0.950
Q05639	Elongation factor 1-alpha 2	EEF1A2	100,940	7.45E-04	0.954

**Compared to untreated cells.

A total of 3715 proteins were differentially expressed in the differentiated SH-SY5Y neural cells treated with TMC, with 838 proteins sharing the common data sets. When the protein profiles from the TMC treatment were compared with that of the untreated differentiated SH-SY5Y cells, a total of 202 proteins were found to match both protein data sets with a total of 87 proteins exhibiting differential expression that were statistically significant (p < 0.05); of which 53 proteins were upregulated, and 34 proteins were downregulated. The top three proteins that were markedly upregulated in the differentiated SH-SY5Y neural cells treated with TMC were dihydrolipoyl dehydrogenase mitochondrial (DLD), neuroblast differentiation-associated protein (AHNAK) and vimentin (VIM). While the top three proteins that were down-regulated include citrate synthase mitochondrial (CS), calnexin (CANX) and transcription intermediary factor-1 beta (TRIM28) were the top 3 down-regulated proteins (Table 2).

**Table 2:** Proteins that were (A) up or (B) down-regulated proteins in differentiated SH-SY5Y neural cells following exposure to TMC.

(A) Upregulated proteins					
Accession	Protein description	Gene symbol	Average mass	p value	fold change
P09622	Dihydrolipoyl dehydrogenase mitochondrial	DLD	54,177	1.26E-03	2.48
Q09666	Neuroblast differentiation-associated protein	AHNAK	629,114	7.64E-03	2.46
P08670	Vimentin	VIM	53,652	5.78E-04	2.46
Q07021	Complement component 1 Q subcomponent-binding protein mitochondrial	C1QBP	31,362	2.16E-02	1.72
P07355	Annexin A2	ANXA2	38,604	2.64E-05	1.68
P35637	RNA-binding protein FUS	FUS	53,426	5.42E-03	1.65
P07237	Protein disulfide-isomerase	P4HB	57,116	1.13E-02	1.62
P21796	Voltage-dependent anion-selective channel protein 1	VDAC1	30,773	2.74E-02	1.61
P38646	Stress-70 protein mitochondrial	HSPA9	73,681	3.03E-02	1.45
P37108	Signal recognition particle 14 kDa protein	SRP14	14,570	3.54E-03	1.40
P35232	Prohibitin	PHB	29,804	2.77E-02	1.38
P18669	Phosphoglycerate mutase 1	PGAM1	28,804	4.96E-02	1.36
P14174	Macrophage migration inhibitory factor	MIF	12,476	8.71E-03	1.35
P24752	Acetyl-CoA acetyltransferase mitochondrial	ACAT1	45,200	8.80E-03	1.34
Q16658	Fascin	FSCN1	54,530	1.52E-03	1.33
P61981	14-3-3 protein gamma	YWHAG	28,303	3.12E-02	1.29
P25705	ATP synthase subunit alpha mitochondrial	ATP5F1A	59,751	3.61E-02	1.29
P62424	60S ribosomal protein L7a	RPL7A	29,996	2.58E-02	1.29
Q9UL46	Proteasome activator complex subunit 2	PSME2	27,402	1.22E-02	1.28
P10809	60 kDa heat shock protein mitochondrial	HSPD1	61,055	1.60E-02	1.26
P49327	Fatty acid synthase	FASN	273,424	3.85E-03	1.26
P14618	Pyruvate kinase PKM	PKM	57,937	5.24E-06	1.25
P62841	40S ribosomal protein S15	RPS15	17,040	1.25E-02	1.23
P30101	Protein disulfide-isomerase A3	PDIA3	56,782	3.88E-04	1.22
P37802	Transgelin-2	TAGLN2	22,391	4.85E-02	1.22
Q9BVA1	Tubulin beta-2B chain	TUBB2B	49,953	8.60E-03	1.21
Q96AG4	Leucine-rich repeat-containing protein 59	LRRC59	34,930	4.98E-05	1.20
P09429	High mobility group protein B1	HMGB1	24,894	4.97E-02	1.20
P08238	Heat shock protein HSP 90-beta	HSP90AB1	83,264	4.33E-03	1.19
P07195	L-lactate dehydrogenase B chain	LDHB	36,639	1.47E-03	1.17
Q13509	Tubulin beta-3 chain	TUBB3	50,433	2.28E-03	1.15
P68371	Tubulin beta-4B chain	TUBB4B	49,831	3.03E-02	1.15
P68032	Actin alpha cardiac muscle 1	ACTC1	42,019	1.10E-03	1.15
P29401	Transketolase	TKT	67,878	3.07E-02	1.14
Q15084	Protein disulfide-isomerase A6	PDIA6	48,121	1.14E-02	1.14
P23246	Splicing factor proline- and glutamine-rich	SFPQ	76,150	3.38E-02	1.14
P0C0S5	Histone H2A.Z	H2AFZ	13,553	8.00E-03	1.14
Q9P0M6	Core histone macro-H2A.2	H2AFY2	40,058	3.71E-02	1.14
Q99623	Prohibitin-2	PHB2	33,296	1.26E-02	1.13
P09936	Ubiquitin carboxyl-terminal hydrolase isozyme L1	UCHL1	24,824	2.16E-02	1.12
Q9BUF5	Tubulin beta-6 chain	TUBB6	49,857	3.45E-02	1.11
P40926	Malate dehydrogenase mitochondrial	MDH2	35,503	6.29E-03	1.10
P00558	Phosphoglycerate kinase 1	PGK1	44,615	2.61E-02	1.10
P15121	Aldose reductase	AKR1B1	35,853	5.42E-03	1.09
P06748	Nucleophosmin	NPM1	32,575	2.26E-02	1.08
P06733	Alpha-enolase	ENO1	47,169	3.04E-02	1.08
P22626	Heterogeneous nuclear ribonucleoproteins A2/B1	HNRNPA2B1	37,430	1.77E-02	1.08
P68363	Tubulin alpha-1B chain	TUBA1B	50,152	4.30E-02	1.07
P36578	60S ribosomal protein L4	RPL4	47,697	3.20E-02	1.06
P23284	Peptidyl-prolyl cis–trans isomerase B	PPIB	23,743	3.08E-02	1.06
P62906	60S ribosomal protein L10a	RPL10A	24,831	2.18E-03	1.04
P62826	GTP-binding nuclear protein Ran	RAN	24,423	7.09E-03	1.03
P52209	6-phosphogluconate dehydrogenase decarboxylating	PGD	53,140	4.33E-02	1.02

(B) Down-regulated proteins					
Accession	Protein description	Gene symbol	Average mass	p value	fold change
O75390	Citrate synthase mitochondrial	CS	51,712	1.46E-02	0.39
P27824	Calnexin	CANX	67,568	6.57E-03	0.55
Q13263	Transcription intermediary factor 1-beta	TRIM28	88,550	3.77E-02	0.56
P46781	40S ribosomal protein S9	RPS9	22,591	1.19E-03	0.58
P08758	Annexin A5	ANXA5	35,937	2.08E-02	0.59
P0DP23	Calmodulin-1	CALM1	16,838	7.20E-03	0.63
P0DP24	Calmodulin-2	CALM2	16,838	7.20E-03	0.63
P0DP25	Calmodulin-3	CALM3	16,838	7.20E-03	0.63
Q99714	3-hydroxyacyl-CoA dehydrogenase type-2	HSD17B10	26,923	1.61E-02	0.64
Q15366	Poly(rC)-binding protein 2	PCBP2	38,580	8.92E-03	0.64
P62701	40S ribosomal protein S4 X isoform	RPS4X	29,598	1.31E-03	0.70
P40227	T-complex protein 1 subunit zeta	CCT6A	58,024	3.96E-05	0.71
Q14194	Dihydropyrimidinase-related protein 1	CRMP1	62,184	2.19E-02	0.75
P12277	Creatine kinase B-type	CKB	42,644	6.30E-03	0.75
P62269	40S ribosomal protein S18	RPS18	17,719	2.63E-04	0.78
O75367	Core histone macro-H2A.1	H2AFY	39,617	5.91E-03	0.79
P45880	Voltage-dependent anion-selective channel protein 2	VDAC2	31,567	1.66E-02	0.8
P62249	40S ribosomal protein S16	RPS16	16,445	2.52E-02	0.86
P52272	Heterogeneous nuclear ribonucleoprotein M	HNRNPM	77,516	1.41E-02	0.86
P39019	40S ribosomal protein S19	RPS19	16,060	4.19E-02	0.86
P40939	Trifunctional enzyme subunit alpha mitochondrial	HADHA	83,000	4.54E-02	0.88
P55072	Transitional endoplasmic reticulum ATPase	VCP	89,322	1.25E-03	0.88
P83731	60S ribosomal protein L24	RPL24	17,779	1.41E-03	0.88
P20962	Parathymosin	PTMS	11,530	4.32E-02	0.91
P19338	Nucleolin	NCL	76,615	2.31E-02	0.91
P14625	Endoplasmin	HSP90B1	92,469	7.57E-03	0.92
P60842	Eukaryotic initiation factor 4A-I	EIF4A1	46,154	1.81E-02	0.92
O75531	Barrier-to-autointegration factor	BANF1	10,059	1.12E-03	0.93
P06744	Glucose-6-phosphate isomerase	GPI	63,147	1.70E-03	0.96
P61978	Heterogeneous nuclear ribonucleoprotein K	HNRNPK	50,976	1.87E-02	0.96
Q99832	T-complex protein 1 subunit eta	CCT7	59,367	2.32E-02	0.96
P30041	Peroxiredoxin-6	PRDX6	25,035	1.25E-02	0.97
P09211	Glutathione S-transferase P	GSTP1	23,356	2.02E-02	0.98

**Compared to untreated cells.

### 3.5 Functional enrichment analysis

**Fig. 4:**
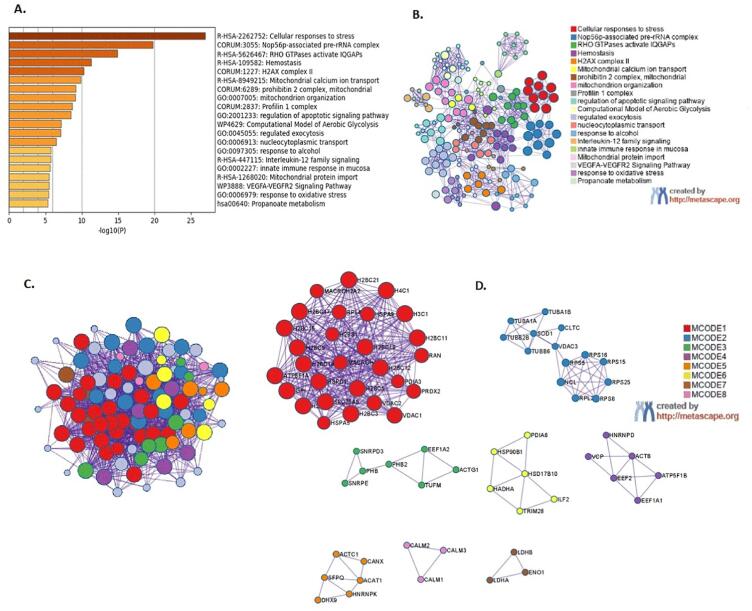
Functional enrichment analysis using Metascape of differentially expressed proteins in the differentiated SH-SY5Y neural cells exposed to 6-OHDA (10 μg/mL) compared to untreated cells. **(A)** Histogram of clustered enrichment ontology categories (GO and KEGG terms) across input gene list, coloured by p-values; **(B)** Network of enriched term of 98 genes that were entered in to this system for analysis. Each term is represented by circle node and the node size is directly proportional to the number of input proteins grouped into each term. The nodes colour denotes its cluster identity. GO terms with a similarity score >0.3 are connected by an edge and the edge thickness represents the similarity score. **(C)** 6-OHDA implicated protein-protein interaction network in differentiated SH-SY5Y neural cells visualized using MCODE algorithm where vicinity densely connected proteins were identified from clustered enrichment ontology terms **(D)** PPI MCODE components identified in the gene lists. Each MCODE network was derived from GO enrichment analysis *(details provided in* Supplementary Tables 1 and 2*).*

Among the 100 proteins significantly regulated by 6-OHDA, 98 proteins were unique proteins and were included in the over-representation functional annotations analysis (Fig. 4A and B; Supplementary Table 1) using the Metascape bioinformatics tool. The analysis revealed 20 GO-biological terms; of which the top five most significant were cellular responses to stress (32 proteins); non-56p-associated pre-rRNA complex (15 proteins), RHO GTPases activate IQGAPs (9 proteins), haemostasis (18 proteins) and H2AX complex II (5 proteins) (Fig. 4B).

Within the KEGG-enriched pathways, we discovered the pathways of neurodegeneration-multiple diseases (hsa02022) was associated with 19 proteins that topped the hit list. The minimal common oncology data elements (MCODE) and protein-protein interaction (PPI) enrichment analysis revealed eight PPI units consisting 59 proteins from which five proteins with respective MCODE scores – VDAC2 (15.5), TUBB2B (10.9), SNRPD3 (5.6), EEF1A2 (6.2) and CALM2 (3.0) – were termed as seed proteins (Fig. 4C and D; Supplementary Table 2). The two major GO-biological processes enriched for the red unit (28 proteins) includes nucleosome assembly (GO:0051170) and nucleosome (GO:0034728); and one KEGG pathway, which was systemic lupus erythematosus (hsa05322).

**Fig. 5:**
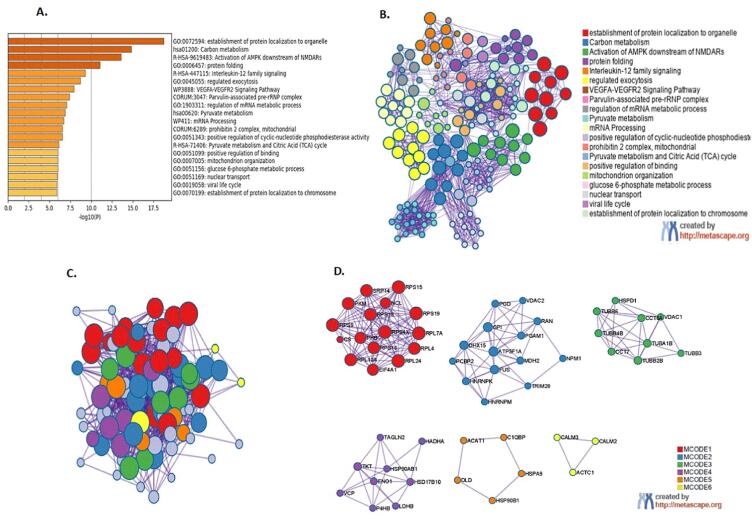
Functional enrichment analysis using Metascape of differentially expressed proteins in the differentiated SH-SY5Y neural cells exposed to TMC (0.1 μg/mL) compared to untreated cells. **(A)** Histogram of clustered enrichment ontology categories (GO and KEGG terms) across input gene list, coloured by p-values; **(B)** Network of enriched term of 87 genes that were entered into this system for analysis. Each term is represented by circle node and the node size is directly proportional to the number of input proteins grouped into each term. The nodes colour denotes its cluster identity. GO terms with a similarity score >0.3 are connected by an edge and the edge thickness represents the similarity score. **(C)** TMC implicated protein-protein interaction network in differentiated SH-SY5Y neural cells visualized using MCODE algorithm where vicinity densely connected proteins were identified from clustered enrichment ontology terms **(D)** PPI MCODE components identified in the gene lists. Each MCODE network was derived from GO enrichment analysis *(details provided in Supplementary Tables 3 and 4).*

In the differentiated SH-SY5Y neural cells treated with TMC, 87 proteins that were significantly *(p < 0.05)* regulated were included in the GO-biological process analysis (Fig. 5A and B; Supplementary Table 3). The top five overexpressed protein clusters included the establishment of protein localisation to organelle (23 proteins), carbon metabolism (12 proteins), activation of AMPK downstream of NMDARs (8 proteins), protein binding (12) and interleukin-12 family signalling (7 proteins). When analysed within the KEGG database, the metabolic pathway (hsa01100) was highly enriched and this involved 21 proteins. The MCODE and PPI enrichment analysis resulted in a network with six PPI units that included 57 proteins from which four seed proteins were identified; which were RPL10A (11.0), PGD (6.8), P4HB (4.4), TUBB6 (8.6) (Fig. 5C and D; Supplementary Table 4). The top three pathway clusters in the red unit (18 proteins) that were overexpressed among 83 GO-BP terms with 2 major GO-BP categories include SRP-dependent co-translational protein targeting to membrane (GO:0006614) and co-translational protein targeting to membrane (GO:0006613); and a Reactome pathway, i.e., L13a-mediated translational silencing of Ceruloplasmin expression (R-HSA-156827).

### 3.6 Venn diagram analysis

**Fig. 6:**
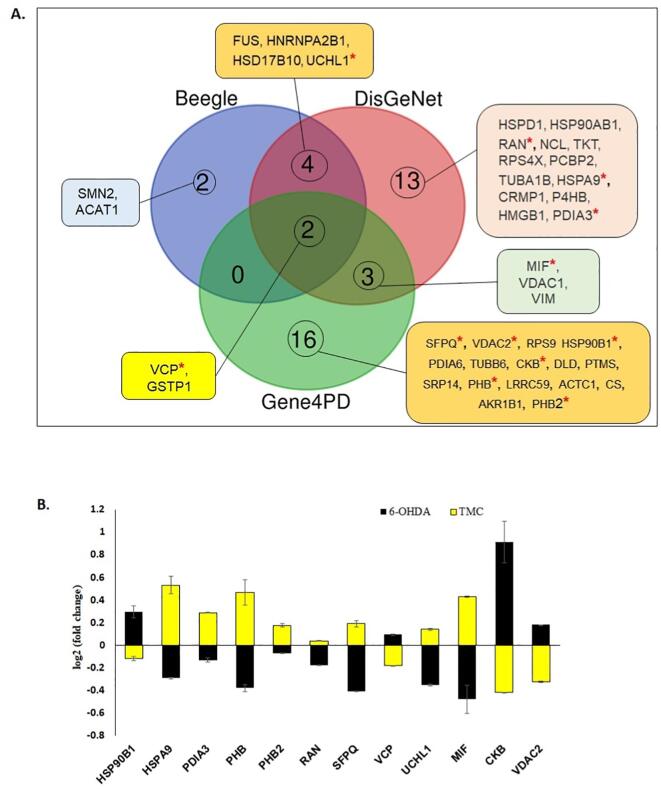
Comparison between PD-associated genes that were differentially expressed in the differentiated SH-SY5Y neural cells following exposure to TMC (0.1 μg/mL) and 6-OHDA (10 μg/mL) using three online PD-gene disease databases (Beegle, DisGeNet and Gene4PD). Forty (40) out of the 87 proteins were found in these three databases. **(A)** Three-set Venn diagram showing the overlap of genes amongst the TMC implicated proteins in the three databases. **(B)** Compares the expression 12 PD-associated genes in the differentiated SH-SY5Y neural cells following exposure to 6-OHDA (black) or TMC (yellow). (For interpretation of the references to colour in this figure legend, the reader is referred to the web version of this article.)

The proteins that were significantly regulated in the differentiated SH-SY5Y neural cells following exposure to TMC or 6-OHDA were compared against three online PD gene-disease repositories (Beegle, DisGeNet and Gene4PD), which contains patient data of genes/proteins that were reported to be involved in the onset or progression of PD. Among the 87 proteins that were significantly regulated in the differentiated SH-SY5Y cells by TMC, eight proteins (FUS, VCP, SMN2, HNRNPA2B1, HSD17B10, ACAT1, UCHL1, GSTP1) matched to the Beegle database; 22 proteins (FUS, CRMP1, GSTP1, HMGB1, HNRNPA2B1, HSD17B10, HSP90AB1, HSPA9, HSPD1, MIF, NCL, P4HB, PCBP2, PDIA3, RAN, RPS4X, TKT, TUBA1B, UCHL1, VCP, VDAC1, VIM) corresponded with the DisGenet database and 21 proteins (AKR1B1, CS, DLD, GSTP1, LRRC59, PDIA6, PHB, PHB2, PTMS, SFPQ, SRP14, TUBB6, VCP, VDAC1, VDAC2, VIM, ACTC1, CKB, MIF, RPS9, HSP90B1) matched the Gene4PD database (Fig. 6A). Two proteins (VCP and GSTP1) were reported in the three databases. Four proteins (FUS, HNRNPA3B1, HSD17B10, UCHl1 were detected in at least two databases, i.e., Beegle & DisGeNet whilst another three proteins (MIF, VDAC1 and VIM) were found in another two databases (DisGeNet & Gene4PD) (Fig. 6A). Some of these proteins exhibited significant differences (p < 0.05) following 6-OHDA treatment (Fig. 6B). Proteins that displayed significant (p < 0.05) expression in the differentiated SH-SY5Y cells following exposure to TMC or 6-OHDA include HSP90AB1, HSPA9, PDIA3, PHB, PHB2, RAN, SFPQ, VCP, UCHL1, MIF, CKB and VDAC2 (Fig. 6B). The proteins modulated by TMC in the differentiated SH-SY5Y cells that correspond to these three PD-associated gene-disease databases were considered significant in PD progression and explored in great detail.

## 4 Discussion

Carotenoids are lipid-soluble and highly unsaturated plant pigments that possess a great ability to cross the blood-brain-barrier. Along with antioxidant and anti-inflammatory effects, carotenoids have a promising role in reducing the risk of neurodegenerative diseases (Lakey-Beitia, Jagadeesh Kumar, Hegde, & Rao, 2019). This study presents the various protective mechanisms of palm derived-TMC on differentiated SH-SY5 Y neural cells exposed to neurotoxin 6-OHDA. The 6-OHDA treatment resulted in a dose-dependent neurotoxicity on differentiated neural cells (Storch, Kaftan, Burkhardt, & Schwarz, 2000). However, TMC attenuated 6-OHDA-induced neurotoxicity, as evidenced by increased cell viability (Fig. 1B) and decreased LDH release (Fig. 1C) from differentiated neural cells. A similar finding was reported by Yi et al, whereby lycopene preincubated dopaminergic SH-SY5Y neuroblastoma cells challenged with 1-methyl-4-phenylpyridinium iodide (MPP^+^), increased cell viability and decreased apoptotic rate (Yi, He, & Wang, 2013). Moreover, lycopene treatment on rat primary hippocampal neurons has been associated with inhibition of mitotic apoptotic pathway and increased viability under neurotoxic condition (Qu et al., 2011a).

Intracellular antioxidant defence enzymes such as SOD and CAT stabilise and deactivate toxic free radicals to stable non-toxic molecules before attacking cellular components (Krishnamurthy & Wadhwani, 2012). The SOD enzyme catalyses the dismutation of superoxide oxygen radicals to less toxic oxygen and hydrogen peroxide molecules (Altobelli, Van Noorden, Balato, & Cimini, 2020), while CAT neutralises hydrogen peroxide generated in cells to water and oxygen products (Glorieux & Calderon, 2017). Our data suggest that TMC exerts its antioxidant mechanisms via two routes: (1) suppression of ROS generation via neutralizing of free radical and (2) upregulation of antioxidant enzyme activity (Fig. 2A–C). These two mechanisms attenuate oxidative stress at the cellular level hence preventing neuronal loss. However, it is crucial to understand if these mechanisms are initiated following the penetration of carotenoids into the cell membrane or at the extracellular region. A previous permeability study revealed that carotenoids interact with membrane lipid bilayer and makes the membrane thicker and solider. These structural changes consequently reduce the membrane permeability to peroxyl and oxygen molecules which protect cells from oxidative stress and cell death (Mostofian, Johnson, Smith, & Cheng, 2020). Besides, the scavenging capacity of carotenoids has been delineated by Liu et al. using the stable free radical 2,2-diphenyl-1-picrylhydrazyl (DPPH) method. According to this report, lycopene or β-carotene in solution demonstrated a significant scavenging capacity. Interestingly, lycopene and β-carotene displayed a synergistic effect in their scavenging capacity when combined with vitamin E and vitamin C compared with individual antioxidant (Liu, Shi, Colina Ibarra, Kakuda, & Jun Xue, 2008). In an experimental model involving ethanol-induced toxicity in embryonic rat hippocampal culture, β-carotene supplementation notably increased neuronal survival via antioxidant activity (Mitchell, Paiva, & Heaton, 1999). We suggest that combining more than one antioxidant like in TMC complex may provide an enhanced antioxidant effectiveness that offers neuroprotective effects in neurons. However, more future studies are needed to understand the underlying mechanism of antioxidant enzyme activation by carotenoids.

Dopamine is a crucial neurotransmitter produced in several areas of the brain, including substantia nigra, hypothalamus and ventral tegmental area (Juárez Olguín, Calderón Guzmán, Hernández García, & Barragán Mejía, 2016). It is a chemical that mediates the “feel-good” feelings (reward, motivation, love) in the brain and motor control and cognitive functions. Degeneration of dopamine-producing neurons, typically the nigral neurons, results in depletion of dopamine, which causes motor problems such as resting tremor, muscle rigidity, loss of postural reflexes, and freezing phenomenon (feet temporarily glued to the ground) (Fahn & Sulzer, 2004). There is compelling data from animal studies that suggest pre-treatment with β-carotene decreases striatal dopamine loss in the substantia nigra in C57 black mice treated with N-methyl-4-phenyl-1,2,3,6-tetrahydropyridine (MPTP) (Perry et al., 1985).

Chu and Kordower have revealed the interconnection between dopamine, TH and α-synuclein. According to this report, the increased expression of α-synuclein was associated with reduced levels of TH and dopamine in sub-threshold degeneration of nigral striatal neurons (Chu & Kordower, 2007). Likewise, the present study displayed that TMC increased the levels *(p < 0.05)* of dopamine (Fig. 3A) and TH (Fig. 3B) in differentiated SH-SY5Y neural cells. In addition, there was a marked reduction of 6-OHDA-induced overexpression of α-synuclein in TMC pre-treated human neural cells (Fig. 3C) (Perez et al., 2002). These findings suggest that the elevated dopamine level in the TMC-treated differentiated SHT-SY5Y cells in the present study may be associated with inhibition of α-synuclein and increased TH levels. Although our findings correlated with Chu and Kordower, we could not confirm if dopamine increase was due to upsurge in dopamine production or reduced catabolism. Hence, future studies should be directed to understand the direct effect of carotenoids on the dopamine biosynthesis pathway.

The DRD2 is a member of the seven-transmembrane and trimeric GTPR (GTP-binding protein-coupled receptor) family and is primarily found in the pre-and post-synaptic dopaminergic neurons at the basal ganglia (striatum and substantia nigra). DRD2 regulates the phosphorylation state of dopamine and TH activity in the synaptic terminals of the substantia nigral. Several studies have reported the connection between polymorphisms in the *DRD2* gene with PD and Schizophrenia (Costa-Mallen et al., 2000, Tan et al., 2000). Our findings revealed that pre-treatment of neural cells with TMC upregulated the *DRD2* mRNA fold change compared to 6-OHDA alone (Fig. 3D). Samad *et al*., Samad, Krezel, Chambon, and Borrelli (1997) have demonstrated a direct mechanistic relationship between DRD2 receptor regulation and retinoids. According to this report, the promoter of the *DRD2* gene contains a functional retinoic acid response element that can be activated with external exposure of retinoids (TA, W, P, & E, 1997). In a subsequent study, Chichili et al. have detected the significant upregulation of the beta-carotene-15,15′-monooxygenase (BCM) enzyme in human retinal pigment epithelial (RPE) cell line (D407) treated with β-carotene (Chichili, Nohr, Schäffer, von Lintig, and Biesalski, 2005). The BCM is crucial in converting the β-carotene into retinoids. The increase in *BCM* mRNA in the RPE cells following exposure to β-carotene suggests the existence of a vitamin A biosynthetic pathway, which may ensure retinoid supply to these cells. Thus, the upregulation of the *DRD2* gene observed in present study following pre-treatment with TMC may be related to the generation of retinoid via the vitamin A pathway that directly affects the expression of the *DRD2* gene. However, more studies are required to confirm this notion.

The expression of many proteins was altered in the differentiated SH-SY5Y cells treated with 6-OHDA or TMC. These changes correlated with over-expression of several molecular and biological processes, as shown in the functional GO analysis (Fig. 4A and A). Forty out of the 87 proteins that were differentially expressed in the differentiated SH-SY5Y cells treated with TMC, were reported to have a close association with the pathogenesis of PD as these were reported in three repositories containing PD gene-diseases, namely Beegle, DisGenet and Gene4PD (Fig. 6A). Twelve of these proteins demonstrated an opposite trend in the 6-OHDA treated neural cells (Fig. 6B). The crucial proteins that TMC mediated caused an overexpression of biological pathways as curated by Metascape.

The top overexpressed functional GO term modulated by TMC on differentiated SH-SY5Y neural cells was the establishment of protein localisation to organelles, which contained 23 proteins. Interestingly, 12 out of these 23 proteins were ribosomal proteins (RPs) (RPL10A, RAN, RPL4, RPL7A, RPL24, RPS4X, RPS9. RPS15, RPS16, RPS18, RPS19, SRP14). The majority of the RPs involved in the ribosome pathway were also present in MCODE1 network proteins (Fig. 4D). The RPs are integral elements of the ribosome machinery responsible for protein synthesis from messenger RNAs (mRNA). The RPs play a pivotal role in ribosome biogenesis (Zhou, Liao, Liao, Liao, & Lu, 2015). Differential regulation of RPs in ribosome biogenesis has been associated with cell differentiation (Bevort & Leffers, 2000), growth (Jorgensen et al., 2004) and proliferation (Volarevic et al., 2000). In addition, inhibition of RPS9 resulted in diminished production of 18S ribosomal RNA and remarkably enhanced p53-dependent morphological differentiation of U343Mga C12:6 glioma cells (Lindström & Nistér, 2010). In the present study, we found TMC caused a statistically significant downregulation of RPS9, RPS4X, RPS16 and RPS19 in human neural cells. The RPs downregulation is directly correlated with increased differentiation in SH-SY5Y cells and this finding is well correlated report by Bevort and Leffers (Bevort & Leffers, 2000).

Moreover, The RAN (Ras-related nuclear protein), a member of the Ras superfamily of small GTPases, was modulated in differentiated SH-SY5Y neural cells treated with TMC. RAN plays a pivotal role in nucleocytoplasmic transport mechanism in both directions by carrying proteins and macromolecules through the nuclear pore complex. The disruption of nucleocytoplasmic transport accompanied by reduced expression of RAN are important leads to the oligomeric Aβ42 formation in Alzheimer’s disease (AD) (Mastroeni et al., 2013). Yasuda, Miyamoto, Saiwaki, and Yoneda (2006) have described that oxidative stress in cells causes a collapse in the RAN gradient and nuclear accumulation of importin alpha that is consequent in either necrotic or apoptotic cell death (Y. Y et al., 2006). Consistent with this report, our data showed that 6-OHDA treatment on neural cells caused significant suppression of RAN, but TMC treatment significantly upregulated the expression of RAN protein. The upregulation of RAN protein mediated by TMC signifies this lipophilic antioxidant's ability to improve the nucleocytoplasmic transport system, which allows the continuous exchange of molecules between nucleus and cytoplasm.

Another critical protein enriched in the establishment of protein localisation to organelle GO term category was the prohibitin 2 (PHB2), which plays a vital role in mitophagy. Mitophagy is a normal cellular physiological process for eliminating dysfunctional or unwanted mitochondria in eukaryotic cells. It maintains cellular homeostasis via coordinating metabolic demand, orchestrating quality control, and protecting cells against impaired mitochondria's destructive effects (Tatsuta & Langer, 2008). A recent study has described the role of PHB2, a highly conserved mitochondria inner membrane scaffold protein, in mediating mitophagy. According to this report, during aggregation of misfolded protein in mitochondria, PHB2 depletion destabilizes PTEN induced kinase 1 (PINK1) and inhibits the mitochondria recruitment of E3 ubiquitin ligase (PRKN), ubiquitin and optineurin, leading to inhibition of mitophagy and cellular apoptosis (Yan et al., 2020). PINK1 and PRKN proteins remove toxic misfolded proteins from mitochondria, and mutations in these genes have been associated with PD (Pickrell & Youle, 2015). Our data showed that TMC treatment on differentiated neural cells tremendously elevated the PHB2, that was significantly suppressed in 6-OHDA exposed cells. These findings indicate that failure in mitophagy may be one of the mechanisms that caused apoptosis in neural cells exposed to 6-OHDA. The elevation of PHB2 by TMC augments proper cellular energy homeostasis regulation by removing damaged mitochondria via mitophagy.

The proteomic analysis also revealed a significant overexpression of α/β isotypes of tubulins (TUBA1B, TUBB3, TUBB4B, TUBB6, TUBB2B) in TMC treated differentiated neural cells compared to untreated control cells. Tubulins are α/β subunit proteins of microtubules that function in cellular division and maintain morphology, intracellular transport, and differentiation (Binarová & Tuszynski, 2019). A substantial body of evidence suggests a marked difference in the functions between tubulin βI, βII and βIII. Using the specific siRNA to inhibit the expression of each tubulin isotype in neuronal cells, this study showed that tubulin βI is vital for cell viability and βII for neurite outgrowth. In comparison, tubulin βIII was important in protecting neural cells against reactive oxygen species and free radical-induced oxidative stress (Guo, Walss-Bass, & Ludueña, 2010). Nevertheless, compelling data from a recent study has suggested that mutations of αβ-tubulin dimers are behind the cognitive impairment in neurodegenerative diseases such as PD and AD (Keays et al., 2007). Moreover, reduced microtubule mass, a higher fraction of unpolymerized tubulin and disruption in microtubule-mediated signalling pathway were indicated in fibroblasts collected from familial and idiopathic PD patients (Cartelli, Goldwurm, Casagrande, Pezzoli, & Cappelletti, 2012). Hence, the significant upregulation of tubulin α/β heterodimer by TMC in neural cells may promote a continuously stable cytoskeletal structure, neurite outgrowth and increase cell viability. These alterations may pave the way to a prolonged life span of neural cells and prevent neurodegenerative diseases from setting in.

Protein disulphide isomerase (PDI) is a versatile redox chaperone located at the endoplasmic reticulum (ER). The critical function of PDI chaperone protein is isomerisation, formation and rearrangement of protein disulphide bonds which offer an additional pathway to maintain the native protein conformation (Perri, Thomas, Parakh, Spencer, & Atkin, 2016). PDI is stimulated during ER stress and is accountable for cellular defence against general protein misfolding via chaperone activity. PDI progressively degrades the accumulated misfolded proteins by translocating to the cytosol via ER association degradation (ERAD). Subsequently, these toxic proteins are destined to be eliminated by the ubiquitin-proteasome system (UPS) (Lee et al., 2010). Besides that, PDI is also necessary in maintaining the cellular redox environment of ER by catalysing the disconnection of non-native disulphide bonds (reduction) and subsequently establishing a correct pairing of cysteines (oxidation) in order to shape the native disulphide bonds (Schwaller, Wilkinson, & Gilbert, 2003). Interestingly, we found that the PDI family proteins such as P4HB (PDIA1), PDIA3 and PDIA6 were strikingly upregulated in differentiated neural cells following treatment with TMC. The increased expression of these PDI chaperone proteins may have contributed to the increased folding capacity in ER and essential effect on regulating the cytoskeleton reorganization of the neuronal cells. An expanding body of evidence has reported that PDI controls cytoskeletal reorganization via forming a disulphide bond with β-actin during adhesion of MEG-01 cells to the extracellular matrix by the integrin-dependent signalling response (Sobierajska et al., 2014). Moreover, a previous microarray study has shown that TMC treatment on HUVECs (human umbilical vein endothelial cells) significantly regulated genes involved in rapid remodelling of protein cytoskeleton, cell-matrix adhesion and matrix reorganization (Dembinska-Kiec et al., 2005). Considering the published data and our findings, we suggest that TMC mediated PDI proteins in neural cells may profoundly affect cell structure and morphology maintenance via cytoskeleton reorganization and cell-cell adhesion.

Heat shock proteins (HSP) are critical regulators of neurons' physiological processes, including protein chaperone and folding, synaptic transmission, ER stress response and cell death response (Bross & Fernandez-Guerra, 2016). Emerging studies have associated the depletion HSPs in neurons with the destruction of brain tissues in PD and AD (Dukay et al., 2019, Stetler et al., 2010). Our study showed a striking overexpression of HSPA9 (HSP70), HSPD1 (HSP60) and HSP90AB1 (HSP90β) in differentiated neural cells following overnight treatment with TMC. The HSPA9 or Mortalin is a mitochondrial chaperone protein that functions as a neuronal sensor and plays a vital role as a quality regulator of proteins translocated into the mitochondria (Ferré, Thouard, Bétourné, Belenguer, Miquel, Peyrin, & Szelechowski, 2020). The association of HSPA9 with imported precursor proteins found in the import channel into the mitochondria implied that HSPA9 is accountable for the unfolding of proteins during their transportation (Jebara, Weiss, & Azem, 2017). This process is crucial as the import channel is narrow (approximately between 20A and 26A) for the movement of folded proteins into the mitochondria (Schwartz & Matouschek, 1999). Nevertheless, convincing data from recent studies suggested that the increased level of HSPA9 contributed to significant neuroprotective effects via upkeeping the mitochondria membrane potential (Qu et al., 2012), and protection against ROS induced lipid peroxidation (Qu et al., 2011b). In light of this, we have also shown that 6-OHDA induced oxidative stress on differentiated SH-SY5Y neural cells demonstrated significant suppression of the HSPA9 (Fig. 6b). As such, oxidative stress-induced neuronal death may be the consequence of suppressing mitochondrial respiration and mitochondrial targeting to synapses exacerbated by depletion of HSPA9 (Zhu et al., 2013). Hence, the effect of TMC in upregulating the HSPA9 expression confers the preservation of mitochondria dynamics and functions in the synapses and axons that promote neuronal survival. On the other hand, the HSPD1, in conjunction with HSP10, is responsible for folding the nascent polypeptide from the unfolded liner chain form following the translocation from the cytoplasm into the mitochondria (Vabulas, Raychaudhuri, Hayer-Hartl, & Hartl, 2010). In humans, a mutation in gene encoding HSPD1-HSP10 chaperonin complex has been found to impair the folding of mitochondrial superoxide dismutase (SOD2) protein despite unchanged SOD2 transcription level (Bie et al., 2016). Indeed, mild deficiency of HSPD1 mainly affects myelination of neurons, whereas more severe decreases in HSPD1 would predominantly affect all tissue and not be compatible with life (Bross, Magnoni, & Sigaard, 2012). The HSP90AB1 (heat chock protein 90 kDa alpha, class B, member 1) is a molecular chaperone of HSP multifamily that is involved in the binding of client proteins, support in efficient protein folding, and maintaining protein stability (Csermely, Schnaider, Soti, Prohászka, & Nardai, 1998). The client proteins interact sequentially with HSP90, HSP70/HSP40 complex and several other co-chaperones, including p23, immunophilins, and HSP 90 ATPase inhibitor Hop dependent on the type of protein or type of damage (Haase & Fitze, 2016). Several studies have pointed out that HSP90 forms crosslinks with actin filaments (KoYASU et al., 1986) and interacts with cytoskeleton structures of neuronal cells to maintain stability and utilize for transport purposes (Quintá, Maschi, Gomez-Sanchez, Piwien-Pilipuk, & Galigniana, 2010). HSP90 promotes the tight packing of the branched actin filaments via induction of N-WASP (neuronal Wiskott-Aldrich Syndrome Protein) and Arp2/3 complex (Park, Suetsugu, Sagara, & Takenawa, 2007). The upregulation of HSP proteins in differentiated SH-SY5Y neural cells in the present cell-based study shows that TMC has many molecular pathways to execute its neuroprotective effects.

## 5 Conclusions

Differentiated SH-SY5Y neural cell pre-treated with TMC and subsequently exposed to 6-OHDA restored the cell viability, LDH enzyme and endogenous antioxidant enzymes (SOD, CAT). Moreover, TMC ameliorated the dopamine biosynthesis and upregulated the DRD2 gene expression in the differentiated neural cells compared to the controls. In proteomic studies, TMC exhibited substantial changes on crucial proteins, such as ribosomal proteins, α/β isotypes of tubulins, protein disulphide isomerases (PDI) and heat shock proteins (HSP) in differentiated human neural cells. We propose that TMC is a potent antioxidant pigment with multiple neuroprotective effects on differentiated SH-SY5Y neural cells.

## Declaration of Competing Interest

The authors declare the following financial interests/personal relationships which may be considered as potential competing interests: Ammu Kutty Radhakrishnan reports financial support was provided by Malaysian Palm Oil Board. KR is employed by the Malaysian Palm Oil Board, who provided the research grant to carry out this study.
