# Supplementary material for: Unravelling the neuroprotective mechanisms of carotenes in differentiated human neural cells: Biochemical and proteomic approaches
Source: Food Chem (Oxf). 2022 Feb 14;4:100088. doi: 10.1016/j.fochms.2022.100088 (PMC8991711; doi:10.1016/j.fochms.2022.100088)
Supplement: Supplementary data 2 [file mmc2.pdf]

MS ISO/IEC 17025 TESTING SAMM NO. 364

ExcelVite

CERTIFICATE OF ANALYSIS

EVTene™ 20%

NATURAL MIXED-CAROTENE COMPLEX 20% OIL CONCENTRATE

Our Reference	:	Sam130917_1 / IMU / 0001
COA No.	:	B1_200229_1/6
Sampling Date	:	7-Jan-2016
Released Date	:	13-Jan-2016
Manufacturing Date	:	7-Jan-2016
Best Before Date	:	6-Jan-2019
Batch No.	:	B1/20/0229_1_070116
Country of Origin	:	Malaysia
Plant Genus & Species	:	*Elaeis guineensis*
Plant Part Used	:	Palm Fruits

	Analysis	Test Method	Principle of Method	Specification	Test Results
1	Physical Appearance*			Opaque Reddish Oil Concentrate	Complies
2	Mixed Carotene, % wt/wt	EvTM_0031	UV-VIS	20.0 Min	20.4
3	Moisture, % wt/wt	AOCS Ca 2e-84, 5th Edition (2003)		1.00 Max	0.07
4	Peroxide Value, meq/kg*	AOCS Cd 8-53, 5th Edition		10.0 Max	2.2
5	Lead (Pb), ppm*	AOCS Ca 15-75, 5th Edition		2.0 Max	< 2.0
6	Heavy Metals (as Pb), ppm*	Japanese Specification & Standards for Food Additives 8th Edition – Heavy Metal Limit Test (HMLT)		10.0 Max	< 10.0
7	Total Viable Aerobic Count, cfu/g**	BP 2014/Volume IV/Appendix XVI B		NMT 1000	< 10
8	Total Combined Molds & Yeast Count, cfu/g**	BP 2014/Volume IV/Appendix XVI B		NMT 100	< 10

Certified By

Name : Ms. Chung Yoke Tying
Position : QC Section Head
IKM Registered Number : M/2586/5261/08
Issued Date : 13th September 2017

EXCELVITE SDN. BHD. 1060267-D

The company is a Recipient of
IKM Laboratory Excellence Award
(Since Year 2007)
IKM Laboratory Excellence Silver Award
(Consecutives 10 Years: 2007 – 2016)

This certificate shall not be reproduced except in full, without written approval of the Quality Control Department

Remarks:
1. All samples tested as per received.
2. Tests marked with * in this report are not SAMM accredited.
3. Tests marked with ** in this report are SAMM accredited. The test result had been approved and endorsed by SAMM approved signatory under microbiology section.
4. EvTM refers to the ExcelVite Validated In-House Method.
5. NMT refers to "Not More Than".
6. The above product is verified Non-GMO by The Non-GMO Project (USA).

This Certificate of Analysis is not construed as a warranty. Customer is responsible to carry out their own analyses necessary to determine the suitability of the product described above for the intended use by the customer. It is the customer's responsibility to verify the batch number of the product received with the numbers contained on this report.

ExcelVite Sdn. Bhd. (No. 1060267-D)
Lot 56442, 7 ½ Mile, Jalan Ipoh/Chemor, 31200 Chemor, Perak, Malaysia. Tel: +60 (05) 2014 192 | Fax: +60 (05) 2014 213 |
www.excelvite.com | info@excelvite.com

SAFETY DATA SHEET

Issued Date	August 2016	Effective Date	August 2016
5th Edition		Review Date	August 2019

SECTION 1: PRODUCT AND COMPANY IDENTIFICATION

PRODUCT NAME: EVTene™ 20%

PRODUCT CLASSIFICATION: Natural Mixed-Carotene Complex 20% Oil Concentrate. Predominantly of ***alpha-carotene, beta-carotene, gamma-carotene and lycopene***. Listed by the FDA as GRAS Nutrient/ Dietary Supplement

Manufacturer Contact: ExcelVite Sdn. Bhd.
Lot 56442, 7 ½ Mile,
Jalan Ipoh / Chemor,
31200 Chemor, Perak,
Malaysia.

Telephone Number: +60(5) 201 4192
(Same number to be used in emergency)

Fax Number: +60(5) 201 4213

Email Address: info@excelvite.com

Marketing & Sales Contact: ExcelVite Inc.
21 Balmoral Court,
Talmadge Village,
Edison New Jersey 08817
USA.

Telephone Number: +1(732) 906 1901
(Same number to be used in emergency)

Fax Number: +1(732) 909 2127

This information is believed to be accurate and represents the best information currently available to us. However we make no warranty of merchantability or any other warranty, express or implied, with respect to such information, and we assume no liability resulting from its use. Users should make their own investigation to determine the suitability of the information for their own particular purposes.

SECTION 2: HAZARDS IDENTIFICATIONS

Classification under Regulation (EC) No 1272/2008 (CLP)	No Classification
Classification of the substances according to Directive 67/548/EEC	No Classification
Most important adverse physicochemical, human health and environmental effective	Substance is not classified as hazardous
Label Element	Not relevant

SECTION 3: COMPOSITION / INFORMATION ON INGREDIENTS

Ingredients Name	Percent (%)	CAS Number
**Total Mixed-Carotene Complex**		
➢ **Alpha – Carotene**	20 - 22	7488-99-5
➢ **Beta – Carotene**		7235-40-7
➢ **Gamma – Carotene**		472-93-5
➢ **Lycopene**		502-65-8
**RBD Palm Olein (Monoglyceride/Diglyceride/Triglyceride)**	78 - 80	Not Noted

Remark: The above data is average analytical results accumulated over the past years.

**Molecular Formula & Molecular Weight**	***Carotene***	$C_{40}H_{56}$	536.88
**Isomeric Forms**	Approximately 34% typical cis-isomers and 66% trans-isomers		

SECTION 4: FIRST AID MEASURES

**Eye**	Flush eyes with large amount of water. If uncomfortable persists, consult or seek medical advice.
**Skin**	Remove contamination clothing. Wash with soap and water
**Inhalation**	Remove to fresh air. If respiratory symptoms occur, obtain medical attention immediately.
**Ingestion**	If overdosed, consult a physician immediately

SECTION 5: FIRE FIGHTING MEASURES

**Flammable**	No
**Fire and Explosion**	Not Noted
**Flash Point**	280°C - 290°C
**Lower Explosive Limits (%)**	Not Applicable
**Upper Explosive Limits (%)**	Not Applicable
**Extinguishing Media**	Dry Chemical; Carbon Dioxide; Foam; Water
**Special hazards arising from the chemical**	No particular hazard known
**Advice for fire-fighters**	Wear proper protective clothing and equipment<br>Wear a self-contained breathing apparatus

SECTION 6: ACCIDENTAL RELEASE MEASURES

CONTAIN ALL SPILLS AND LEAKS TO PREVENT DISCHARGE INTO THE ENVIRONMENT.

**Small Spills**	Spills should be taken up with suitable absorbent and placed in containers. Spill area can be washed with water, collect wash water for approved disposal. Do not flush to storm sewer or water way.
**Large Spills**	Recover spills for reprocessing or approved disposal methods.
**Waste Disposal Method**	In accordance with existing federal, state and local environment regulations.

SECTION 7: HANDLING AND STORAGE

Storage Temperature	Avoid overheating or freezing.
Handling and Storage	Keep in tightly closed containers. Store in cool dry place. Protect from exposure to moisture, heat and direct sunlight.
Other Precautions	Avoid contact. Product may stain.

SECTION 8: EXPOSURE CONTROL / PERSONAL PROTECTION

**Exposure Limits**	
➢ **ACGIH TLV**	Not Applicable
➢ **OSHA PEL**	Not Applicable
**Conditions under which preparation required**	Normal work conditions under subdued light.
**Eye Protection**	Recommended during handling and use. Safety glasses are optional.
**Protective Gloves**	Recommended during handling and use.
**Respiratory Protection**	NIOSH approved mask or respirator.
**Ventilation**	Adequate Ventilation
**Wash Requirements**	Wash with water and soap.
**Other Protective Equipment**	Safety shower and eye bath.

SECTION 9: PHYSICAL AND CHEMICAL PROPERTIES

**Physical Form**	Viscous Oil Concentrate
**Odor and Appearance**	Opaque Reddish Vegetable Oil Concentrate with Typical Odor
**Boiling Point**	Not Noted
**Melting Point**	Not Noted
**Density @ 60°C, g/cm^3**	0.9400
**pH in 1% solution**	Not Significant
**Percentage Volatile By Weight**	Not Noted
**Viscosity @ 75°C, cps**	918
**Evaporation Rate**	Not Noted
**Solubility In Water**	Insoluble
**Vapor Density**	Not Noted
**Vapor Pressure (mm Hg)**	Not Noted
**Solvent Solubility**	Soluble In Oil and Fats. Partially Soluble in Alcohol. Soluble in Chloroform, Iso-Octane, Hexane, Tetrahydrofuran
**Auto flammability**	Not Applicable

SECTION 10: STABILITY AND REACTIVITY

**Stability**	Blanketed with nitrogen<br>Stable under conditions of cool and dry unopened containers. Avoid damp and humid conditions.
**Incompatibilities**	Strong oxidizing agent<br>Strong acid / alkali<br>Strong light / heat / air
**Hazardous Decomposition Products**	Not To Be Expected
**Hazardous Polymerization**	Not To Be Expected

SECTION 11: TOXICOLOGICAL INFORMATION

Short Term Effects of Exposure

**Route of Entry**	☒ Eye Contact ☒ Skin Contact ☒ Inhalation ☒ Ingestion
**Acute Effects-Eye Contact**	Low order of toxicity
**Acute Effects-Skin Contact**	Low order of toxicity
**Acute Effects-Inhalation**	Low order of toxicity
**Acute Effects-Ingestion**	Non-Hazardous

Long Term Effects of Exposure

**Effects of Chronic Exposure**	None
**Target Organ**	Not Applicable

Special Health Effects

**Carcinogen (OSHA guidelines)**	No

Product Toxicology

**Oral Toxicity**	Low order of toxicity
**Dermal Toxicity**	Non-hazardous
**Inhalation Toxicity**	Not Noted
**Eye Irritation**	Physical form may cause irritation

Chronic Effects and Medical Conditions Aggravated by Overexposure:

Chronic Effects and Medical Conditions Aggravated by Overexposure to This Product Have Not Been Established.

IF ANY SYMPTOMS, PLEASE CONSULT A PHYSICIAN.

CARCINOGEN: OSHA, NTP, OR IARC DOES NOT CONSIDER THIS PRODUCT AS CARCINOGEN.

SECTION 12: ECOLOGICAL INFORMATION

Not Noted

SECTION 13: DISPOSAL CONSIDERATIONS

This product is not regarded as hazardous waste.

**Waste Disposal Method**	In accordance with existing federal, state and local environment regulations.

SECTION 14: TRANSPORT INFORMATION

**Hazard Class**	Non-hazardous.<br>Listed by the FDA as GRAS nutrient / dietary supplement. European Communities Code: E160a (ii) – Plant Carotenes.
**Shipping Name**	EVTene™ 20%
**Label Information**	Listed on labels are the Product Name, Batch Number, Manufacturing / Expired Date and Net Weight.
**Harmonized Tariff Code**	3203.00.000

SECTION 15: REGULATORY INFORMATION

1 Listed by the FDA as GRAS nutrient / dietary supplement. European Communities Code: E160a (ii) – Plant Carotenes.

2 **BSE / TSE Information** : EVTene™ 20% does not contain any substance or component of bovine, ovine or caprine in the composition.

It also stated that:

- Throughout the manufacturing process of this raw material, no substance or reagent of the bovine, ovine and caprine origin have been used.
- The raw material concerned has not been exposed to risk of environmental contamination from substances of bovine, ovine or caprine origin, during the said manufacturing process.

3 **Vegan / Vegetarian Status** : EVTene™ 20% is suitable for vegan / vegetarian.

4 **Allergen Information** : EVTene™ 20% is free from the "8 Big" allergens – peanut, milk or milk by-product, wheat or gluten, tree nuts, soy, fish and shellfish.

Remarks: The "8 Big" allergen is defined in the Food Allergy Labeling & Consumer Protection Act of 2004 (FALCPA).

5 **GMO Status** : EVTene™ 20% is a 100% NON-GMO product.

SECTION 16: OTHER INFORMATION

**SDS Document Number**	QA_MSDS P001C01
**SDS Issued Date**	August 2016
**SDS Effective Date**	August 2016
**SDS Review Date**	August 2019
**SDS Edition**	5
**SDS Revision Number**	4
**SDS Replace**	Document dated August 2015, 4th Edition.

**************** ADDITIONAL INFORMATION *****************

THIS INFORMATION ON THIS SHEET IS ASSEMBLED BY THE MANUFACTURER BASED ON ITS OWN STUDIES AND OTHERS. THE MANUFACTURER MAKES NO WARRANTIES, EXPRESS OR IMPLIED, AS TO THE ACCURACY, COMPLETENESS, OR ADEQUACY OF THE INFORMATION CONTAINED HEREIN.THE MANUFACTURER SHALL NOT BE LIABLE TO ANYONE FOR ANY DIRECT SPECIAL OR CONSEQUENTIAL DAMAGES ARISING OUT OF OR IN CONNECTION WITH THE ACCURACY, COMPLETENESS, ADEQUACY, OR FURNISHING OF SUCH INFORMATION.
